# Asymptomatic SARS-CoV-2 infection: A systematic review and meta-analysis

**DOI:** 10.1073/pnas.2109229118

**Published:** 2021-08-10

**Authors:** Pratha Sah, Meagan C. Fitzpatrick, Charlotte F. Zimmer, Elaheh Abdollahi, Lyndon Juden-Kelly, Seyed M. Moghadas, Burton H. Singer, Alison P. Galvani

**Affiliations:** ^a^Center for Infectious Disease Modeling and Analysis, Yale School of Public Health, New Haven, CT 06520;; ^b^Center for Vaccine Development and Global Health, University of Maryland School of Medicine, Baltimore, MD 21201;; ^c^Agent-Based Modelling Laboratory, York University, Toronto, ON M3J 1P3, Canada;; ^d^Emerging Pathogens Institute, University of Florida, Gainesville, FL 32610

**Keywords:** asymptomatic fraction, presymptomatic, silent transmission, novel coronavirus, comorbidity

## Abstract

Quantification of asymptomatic infections is fundamental for effective public health responses to the COVID-19 pandemic. Discrepancies regarding the extent of asymptomaticity have arisen from inconsistent terminology as well as conflation of index and secondary cases which biases toward lower asymptomaticity. We searched PubMed, Embase, Web of Science, and World Health Organization Global Research Database on COVID-19 between January 1, 2020 and April 2, 2021 to identify studies that reported silent infections at the time of testing, whether presymptomatic or asymptomatic. Index cases were removed to minimize representational bias that would result in overestimation of symptomaticity. By analyzing over 350 studies, we estimate that the percentage of infections that never developed clinical symptoms, and thus were truly asymptomatic, was 35.1% (95% CI: 30.7 to 39.9%). At the time of testing, 42.8% (95% prediction interval: 5.2 to 91.1%) of cases exhibited no symptoms, a group comprising both asymptomatic and presymptomatic infections. Asymptomaticity was significantly lower among the elderly, at 19.7% (95% CI: 12.7 to 29.4%) compared with children at 46.7% (95% CI: 32.0 to 62.0%). We also found that cases with comorbidities had significantly lower asymptomaticity compared to cases with no underlying medical conditions. Without proactive policies to detect asymptomatic infections, such as rapid contact tracing, prolonged efforts for pandemic control may be needed even in the presence of vaccination.

## Significance

Asymptomatic infections have been widely reported for COVID-19. However, many studies do not distinguish between the presymptomatic stage and truly asymptomatic infections. We conducted a systematic review and meta-analysis of COVID-19 literature reporting laboratory-confirmed infections to determine the burden of asymptomatic infections and removed index cases from our calculations to avoid conflation. By analyzing over 350 papers, we estimated that more than one-third of infections are truly asymptomatic. We found evidence of greater asymptomaticity in children compared with the elderly, and lower asymptomaticity among cases with comorbidities compared to cases with no underlying medical conditions. Greater asymptomaticity at younger ages suggests that heightened vigilance is needed among these individuals, to prevent spillover into the broader community.

COVID-19 surveillance provides real-time information about the epidemiological trajectory of the pandemic, informing risk assessments and mitigation policies around the world. Given that COVID-19 surveillance systems predominantly rely on symptom-based screening, the prevalence of asymptomatic infection is often not fully captured. Cross-sectional surveys, such as mass testing once an outbreak is identified, do not distinguish the truly asymptomatic from the presymptomatic. Often, the follow-up period after testing is too brief to ascertain whether patients subsequently develop symptoms. The percentage of silent infections identified by such studies is thus context specific, as it depends on the setting, phase of the epidemic, and efficiency of contact tracing. By contrast, the prevalence of truly asymptomatic infections should be stable across similar demographic settings, regardless of epidemiological trajectory and contact tracing.

Compounded by ambiguities about the different clinical manifestations of the disease, which can lead to misinterpretation of clinical and epidemiological studies (1), there have been substantial aberrations in reports and media coverage claiming the asymptomatic percentage to be as low as 4% (2, 3) or as high as 80 to 90% (4, 5). Similarly, the US Centers for Disease Control and Prevention guidelines for COVID-19 pandemic forecasting offer wide bounds for the asymptomatic percentage, ranging from 10 to 70% (6).

Previous meta-analyses of 41 studies (7), 13 studies (8), and 79 studies (9) estimate pooled asymptomaticity ranging from 16 to 20%. Two methodological issues limit the accuracy of these studies. First, pooled asymptomaticity reported in these studies is likely biased downward because they did not account for study designs which have a higher representation of cases experiencing symptoms (10). Second, one of the meta-analyses (7) did not consider biases in reported asymptomaticity that can arise from inadequate longitudinal follow-up. Studies that assess the symptom profile only at the time of testing or do not follow up symptoms for a sufficiently long time period cannot distinguish presymptomatic from asymptomatic infection, overestimating those that are truly asymptomatic.

Accurate estimates of true disease prevalence, including asymptomatic infections, are essential to calculate key clinical parameters, project epidemiological trajectories, and optimize mitigation measures. Clinical evidence indicates that viral loads among asymptomatic and symptomatic infections may be comparable (11–15). Unaware of their risk to others, individuals with silent infections are likely to continue usual behavior patterns. Accounting for silent severe acute respiratory syndrome coronavirus 2 (SARS-CoV-2) infections in the assessment of disease control measures is necessary to interrupt community transmission (16). Although the discrepancy between reported incidence and seroprevalence gives a sense of the extent of asymptomaticity, not all symptomatic cases are reported, and not all asymptomatic cases (for instance, those identified on the basis of exposure) are missed. Consequently, it is not sufficient to simply compare the reported cases to results from seroprevalence studies. We therefore conducted a systematic review and meta-analysis of COVID-19 literature reporting laboratory-confirmed infections to estimate the percentage of SARS-CoV-2 infections that are truly asymptomatic. We also investigated differences in asymptomaticity with respect to age, sex, comorbidity, study design, publication date, duration of symptom follow-up, geographic location, and setting.

## Results

**Fig. 1.**
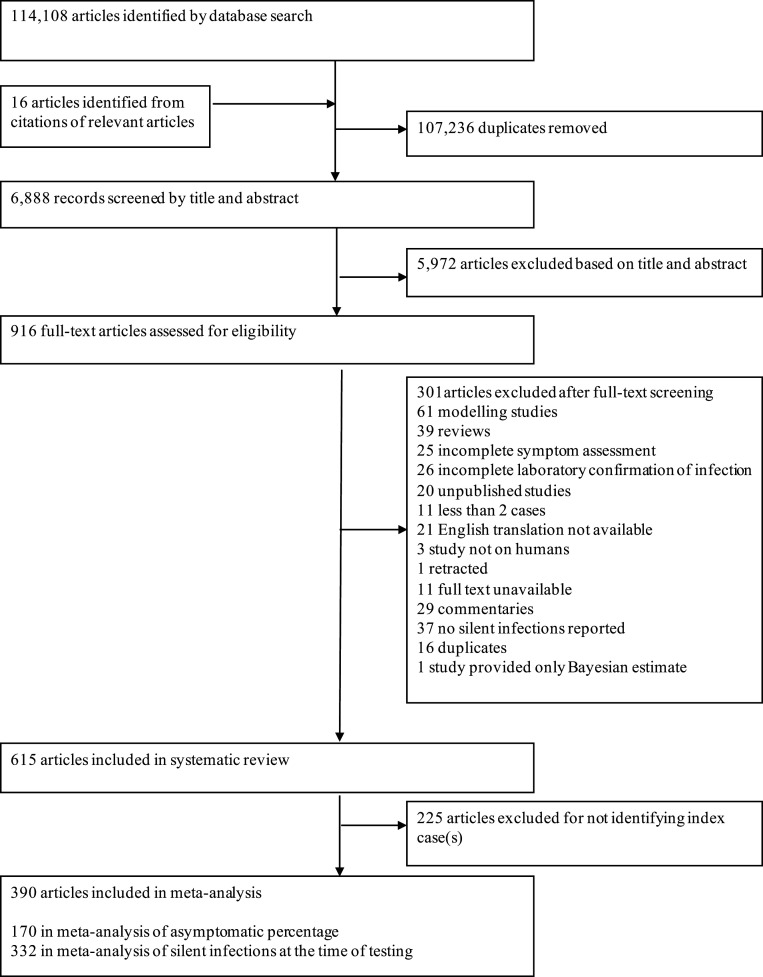
Preferred Reporting Items for Systematic Reviews and Meta-Analyses (PRISMA) flow diagram showing the numbers of studies screened and included in the meta-analysis.

We identified a total of 114,124 abstracts based on our search criteria. After excluding duplicate and irrelevant studies, we used 390 in our meta-analyses (Fig. 1 and *SI Appendix*, Table S2). Most studies were conducted in China (*n* = 104, 27%), followed by the United States (*n* = 74, 19%), Italy (*n* = 21, 5%), and South Korea (*n* = 13, 3%). These studies included a total of 104,058 laboratory-confirmed COVID-19 cases, of which 25,050 exhibited no symptoms at the time of testing and 7,220 remained asymptomatic. We identified 170 studies that reported asymptomatic infections (11–13, 17–183), 332 studies that reported silent infections at the time of testing (10–12, 14, 17–20, 23–27, 31, 32, 35–40, 42–44, 46, 47, 49, 50, 52, 53, 56–58, 60–66, 68, 69, 73–75, 77–79, 81, 84, 87, 90–94, 97, 99, 101, 103, 104, 106, 111, 113–116, 118, 119, 121–123, 125, 127, 128, 131, 133, 135, 137, 138, 140, 143, 145, 146, 148–152, 154, 156, 158, 160–163, 166–170, 172–174, 176, 177, 179, 180, 182–405), and 143 that delineated presymptomatic and asymptomatic infections by following-up with those silently infected (11–13, 17–20, 22–29, 31–33, 35–40, 42–44, 46–54, 56–70, 72–75, 77–81, 83, 84, 87, 89–94, 96, 97, 99, 101, 103, 104, 106–109, 111–119, 121–125, 127–129, 131, 133, 135–138, 140, 141, 143, 145–156, 158–164, 166–170, 172–174, 176–183). Among the studies that reported follow-up of clinical symptoms after testing, 11.0% reported at time points at 1 wk to 2 wk, 33.8% reported at 2 wk to 3 wk, and 55.2% reported longer than 3 wk. Among the studies that reported asymptomatic infections, 58.8% reported zero index cases, either because cases were identified through a screening design or because the study only reported the cases that were identified through contact tracing. Of the 41.2% studies that reported data on index cases, these included household members, long-term care residents, members of the community, or travelers returning from COVID-19 hotspots (*SI Appendix*, Table S1). A summary of the risk of bias assessment is presented in *SI Appendix*, Table S2. Out of the 170 studies included in the calculation of asymptomaticity, 75 had low risk of bias, 10 had moderate risk of bias, and 85 had serious risk of bias.

**Fig. 2.**
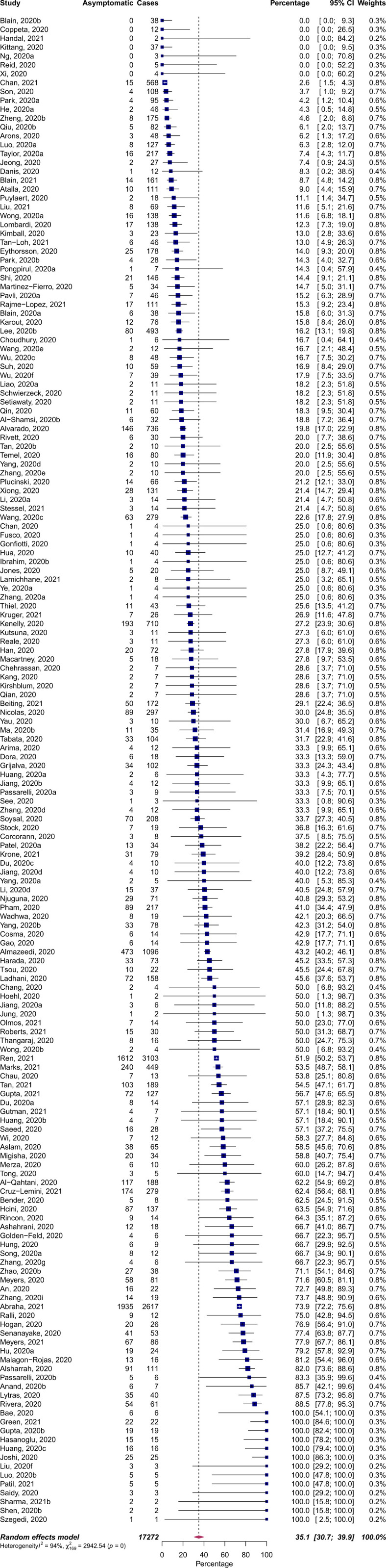
Pooled percentage of laboratory-confirmed COVID-19 cases which remained asymptomatic. Studies that did not report follow-up of silent infections or failed to identify index cases were excluded from the analysis.

The percentage of cases that were truly asymptomatic among laboratory-confirmed cases was 35.1% (95% CI: 30.7 to 39.9%; Fig. 2). By contrast, a larger percentage of cases exhibited no symptoms at the time of testing (42.8%, 95% prediction interval: 5.2 to 91.1%) due to mischaracterization of presymptomatic cases as asymptomatic. To investigate the degree of mischaracterization, we considered a subset of studies that reported symptoms both at the time of testing and a minimum of 7 d after. Within this subset of studies, 31.8% (95% prediction interval: 5.6 to 78.7%) of cases exhibiting no symptoms at the time of testing progressed to develop symptoms. The percentage of truly asymptomatic cases among these studies was therefore 36.9% (95% CI: 31.8 to 42.4%), similar to that estimated for all studies reporting asymptomatic infections.

These estimates were obtained after removing index cases from our calculations, correcting bias toward overrepresentation of symptomatic cases that would lead to underestimation of asymptomaticity. Without excluding index cases, estimates of asymptomatic infections using our two complementary approaches would be 27.8% (95% CI: 24.3 to 31.7%) and 29.4 (95% CI: 25.2 to 33.9%). To evaluate the impact of sample selection bias arising from higher participation among those experiencing symptoms, we next restricted our analysis to 25 studies in which complete screening of every individual present at the setting was performed. The pooled asymptomaticity among this smaller subset of studies was 47.3% (95% CI: 34.0 to 61.0%).

We found a statistically significant trend toward a lower asymptomatic percentage with increasing age (*P* < 0.01; Table 1). In pairwise comparisons, the asymptomatic percentage was significantly lower for the elderly, at 19.7% (95% CI: 12.7 to 29.4%) compared with 46.7% (95% CI: 32.0 to 62.0%) for children (*P* < 0.01). Asymptomaticity also varied across study settings (*P* = 0.03; Table 1). In particular, studies on long-term care facilities reported lower asymptomaticity compared with studies on healthcare facilities (*P* = 0.04) and household transmission (*P* = 0.04). We found no association between asymptomatic percentage and geographic location, study design, follow-up duration, or publication date (Table 1). We found that asymptomaticity in males was similar to that in females (log incidence rate ratio [IRR] 0.09, 95% CI −0.07 to 0.25, *P* = 0.27; *SI Appendix*, Fig. S1). Cases with comorbidities had lower asymptomaticity compared to cases with no underlying medical conditions (log IRR −0.43, 95% CI −0.82 to −0.04, *P* = 0.03; *SI Appendix*, Fig. S2).

**Table 1.** Pooled estimates for percentages of all positive cases which remain asymptomatic stratified by age, gender, publication date, symptom follow-up duration, study design, and study setting

	*n*	Estimate (%)	CI (95%)	*P* value (test of overall effect)
Age class				**<0.01**
Children (0 y to 18 y)	18	46.7	32.0 to 62.0	
Adults (19 y to 59 y)	17	32.1	22.2 to 43.9	
Elderly (≥60 y)	17	19.7	12.7 to 29.4	
Study design				0.10
Population screening	102	38.2	32.0 to 44.8	
Others	68	30.7	24.8 to 37.4	
Publication date				0.18
January–April 2020	27	34.8	23.6 to 47.9	
May–August 2020	69	29.5	24.2 to 35.4	
September–December 2020	50	41.1	31.4 to 51.4	
January–April 2021	24	38.4	25.6 to 53.1	
Symptom follow-up duration				0.07
7 d to 21 d	73	40.6	32.9 to 48.6	
21+ d	90	32.1	27.0 to 37.7	
Setting				**0.03**
Community	39	34.0	25.3 to 43.8	
Healthcare facility	81	38.5	31.6 to 45.9	
Household	18	42.5	30.9 to 54.9	
Long-term care facility	15	17.8	9.7 to 30.3	
Others	17	38.4	23.5 to 55.9	
Geographic location				0.78
China	50	33.6	26.1 to 42.0	
United States	28	33.3	22.6 to 46.1	
Others	92	36.8	30.4 to 43.6	

Stratifications with statistically significant subgroup differences (*P* < 0.05) are in bold.

Egger’s test for asymptomatic percentage was significant (*P* = 0.04; *SI Appendix*, Fig. S3), providing evidence of potential small-study effects. We therefore conducted a sensitivity analysis by excluding studies with relatively small sample sizes (less than 10 infections). The pooled estimate in the restricted meta-analysis (33.1%; 95% CI: 28.0 to 38.5%) was similar to our original estimate, suggesting that our estimates are robust to publication bias.

## Discussion

The SARS-CoV-2 pandemic infected more than 80 million people within a year and is still spreading rapidly despite widespread control efforts. The elements of the global response are similar to those deployed during the SARS-CoV-1 outbreak: detecting new cases through symptom-based surveillance, subsequent testing, and isolation of confirmed cases. In 2002, these measures achieved containment within 8 mo and fewer than 8,500 cases worldwide. Given that the aerosol and surface stability of the two viruses are similar (406), a crucial difference between the two outbreaks could be the role of silent infections in propagating transmission chains. Multiple clinical studies have indicated that viral loads in asymptomatic and symptomatic infections of COVID-19 may be similar (11–14, 354). Furthermore, the presymptomatic phase of SARS-CoV-2 is highly infectious (53), and transmission from those in this phase may be responsible for more than 50% of incidence (16). This is a striking difference from SARS-CoV-1 in which the infectiousness peaked at 12 d to 14 d after symptom onset (407). Although silent infections of SARS-CoV-1 were reported, no known transmission occurred from silently infected or even mildly symptomatic SARS cases.

Since the emergence of COVID-19, there has been much speculation about the silent transmission of the disease. Cross-sectional studies testing exposed individuals who do not exhibit symptoms often conflate asymptomatic infections with those in the presymptomatic phase, leading to substantial overestimation of asymptomatic infection. Longitudinal studies without sufficient follow-up similarly lead to overestimation of asymptomaticity (408). Additionally, inconsistent use of terminology has led to confusion, particularly when distinguishing infections which are silent at the time of testing from those which are truly asymptomatic (4, 5). A previous meta-analysis, for example, incorrectly includes infections in the presymptomatic phase to calculate pooled estimate of asymptomatic percentage (409). By contrast, several studies conducted early in the pandemic reported few asymptomatic infections, primarily due to restrictive testing criteria which focused on testing of severe cases that required hospitalization (410, 411). Inaccuracy in either direction is detrimental for public health. Overestimation of asymptomaticity engenders a perception that SARS-CoV-2 is less virulent, whereas underestimation skews key epidemiological parameters such as infection fatality rate and hospitalization rate upward, leading to suboptimal policy decisions.

To robustly estimate the asymptomatic percentage from studies with varying degrees of methodological vigor, we conducted two separate meta-analyses. In the first analysis, we estimated the asymptomatic percentage as 35.1% (95% CI: 30.7 to 39.9%), by including all studies with a duration of follow-up sufficient to identify asymptomatic infections. In the second analysis, we only included studies that both delineated silent infections at the time of testing and conducted follow-up to distinguish the presymptomatic stage from asymptomatic infections. With this analysis, we estimated the asymptomatic percentage as 36.9% (95% CI: 31.8 to 42.4%). Our estimates have overlapping CIs, which suggests that our pooled analysis is robust to methodological differences in symptom assessment. Our estimates are higher than the 15.6% (95% CI: 10.1 to 23.0%), 17% (95% CI: 14 to 20%), and 20% (95% CI: 17 to 25%) reported by three previous meta-analyses using 41 studies (7), 13 studies (8), and 79 studies (9). In large part, this difference arises because we excluded index cases from our calculation, correcting a bias that leads to underestimation of asymptomaticity. Our estimates of asymptomatic percentage without excluding index cases were 27.8% and 29.4%, for our two approaches. The lower bounds of 24% and 25%, for the two analyses overlaps with the range of the previous largest meta-analysis. Compared with other respiratory infections, the lower bound of our analyses is higher than the 13 to 19% estimated for influenza (412, 413), and the 13% for SARS-CoV-1 (414).

We found that 42.8% (95% prediction interval: 5.2 to 91.1%) of infections were silent at the time of testing. These cases have been incorrectly referred to as asymptomatic in previous studies (4, 5, 189, 239). This rate is context specific, as it is likely influenced by the association between symptomaticity and the time window when an infection is detectable or tested by RT-PCR. Additionally, the proportion of silent infections at the time of testing is highly sensitive to the efficiency of contact tracing. If most contacts are identified and tested swiftly, then nearly all infections will be silent at the time of testing. By contrast, if contact tracing is slow and incomplete, then a larger fraction of individuals will have developed symptoms by the time they are approached for testing, and a smaller proportion of those tested will be symptom-free. Reports of silent infections at the time of testing are also likely impacted by epidemic trajectory largely due to the predominance of recent infections in samples taken during the growth phase, in contrast with a higher proportion of older infections in samples taken during the declining phase. Unbiased measures of asymptomaticity, on the other hand, should be consistent across similar demographic settings, regardless of contact tracing and epidemic trajectory.

Several gaps remain in our understanding of asymptomatic carriage of COVID-19. Particularly, it is unclear why certain infections remain asymptomatic while the majority develop clinical symptoms. Our results indicate that children have greater asymptomaticity compared to the elderly. We also found that cases with comorbidities have lower asymptomaticity compared with cases with no underlying medical conditions. Additionally, studies on long-term care facilities reported lower asymptomaticity compared to other study settings. Given that the risk of severe illness is high among the elderly, the age association identified by our study implies that absence of symptoms may correlate with the tendency of developing milder symptoms. Case severity in SARS-CoV-2 patients has been linked to a cytokine storm which occurs more frequently in elderly patients (415, 416). Genetic (417), environmental risk factors, sex-linked differences (418), and cross-reactive immunity (419) might also contribute, although no studies have unequivocally demonstrated their association with either symptom status or severity.

Higher representation of asymptomatic SARS-CoV-2 infections among younger people has grave implications for control policies in daycares, schools, and universities. Settings with close, extensive contact among large groups of younger individuals are particularly susceptible to superspreader events of COVID-19 which may go undetected if surveillance focuses on symptomatic cases. This close congregation of relatively large groups similarly explains why influenza, mumps, and measles often spread more rapidly in schools and college campuses than in the broader community (420–422). As schools and universities convene in the midst of the COVID-19 pandemic, campus outbreaks are increasingly reported (423). Although COVID-19 severity is lower among young people, campus transmission with a large undetected component could more easily bridge to the rest of the population, fueling local and regional resurgence.

Our meta-analyses are subject to limitations, many related to the unprecedented pace of clinical research since the emergence of COVID-19. First, we found considerable heterogeneity in the percentage of asymptomatic infections. Subgroup analysis revealed that studies with longer follow-up reported lower asymptomaticity. Second, all reports of asymptomatic cases are confounded by the subjective and shifting definition of symptoms. For instance, the list of clinical manifestations associated with COVID-19 has expanded since the initial definitions (424). These changing definitions impact the classification of infections as asymptomatic or silent, and the more limited suite of symptoms initially considered indications of COVID-19 could bias early studies toward higher percentages in these categories. Nonetheless, we found no statistically significant differences in asymptomatic percentage when we stratified studies based on publication date. Third, in the studies included in our meta-analysis, it is possible that early mild symptoms occurring before a positive PCR test might go unrecorded, biasing the studies toward higher asymptomaticity. Fourth, although we corrected for the bias introduced by inclusion of predominantly symptomatic index cases, our estimates are still likely affected by sample selection bias, as participation is expected to be highest among those experiencing symptoms (10). Additionally, factors such as socioeconomic position, occupation, ethnicity, place of residence, internet and technological access, and scientific and medical interest could have contributed to nonrandom enrollment (425). To evaluate the effect of these biases, we calculated the pooled asymptomatic percentage using 25 studies that reported screening of all individuals in the study setting. Asymptomaticity among this smaller subset of studies was 47.3% (95% CI: 34.0 to 61.0%), with CIs that overlap with our primary analysis but the point estimate is higher than the base case CI. We therefore cannot rule out nonrandom sampling as a source of bias for estimation of the asymptomatic percentage.

In our meta-analysis, we excluded 225 studies that did not identify index cases. Additionally, 223 studies reported silent infections at the time of testing but were excluded from analysis of asymptomaticity for not reporting symptom assessment during follow-up for at least 7 d or for not specifying the duration of follow-up. Large-scale longitudinal surveys should prioritize the inclusion of these data to facilitate accurate estimation of the asymptomatic percentage. At minimum, such studies should report the number of index cases among their study participants, the clinical symptom status of individuals at the time of testing, the duration of symptom follow-up, and symptom status during the follow-up. Ideally, studies would additionally provide a full symptom profile both at time of testing and by the end of follow-up, to facilitate reclassification as case definitions are updated.

Estimating the extent of COVID-19 asymptomaticity is critical for calculating key epidemiological characteristics, quantifying the true prevalence of infection, and developing appropriate mitigation efforts. This meta-analysis also establishes a baseline for asymptomaticity, prior to widespread vaccination coverage. Amid concerns that vaccines may be less protective against infection than disease, widespread vaccination coverage may soon lead to a rise in the percentage of infections that present asymptomatically. The high prevalence of silent infections even at baseline, coupled with their transmission potential, necessitates accelerated contact tracing, testing, and isolation of infectious individuals, as symptom-based surveillance alone is inadequate for control.

## Methods

### Definition of Silent, Asymptomatic, and Presymptomatic Infection.

We defined silent infections as laboratory-confirmed COVID-19 cases that did not exhibit any clinical symptoms, including fever, upper respiratory symptoms, pneumonia, fatigue, headache, myalgia, dehydration, or gastrointestinal dysfunction, at the time of testing. Asymptomatic infections include those that continued to exhibit no clinical symptoms during at least 7 d of follow-up after testing. Presymptomatic cases were those that developed clinical symptoms subsequent to initial testing. The presymptomatic stage begins with the start of infectiousness and ends with the onset of symptoms (426).

### Search Strategy and Selection Criteria.

We conducted a systematic review to identify studies reporting laboratory-confirmed COVID-19 cases without symptoms at the time of testing. Our search was inclusive of all studies that provided data regarding cases that were asymptomatic, presymptomatic, or both. We finalized systematic search criteria on May 1, 2020, and study collection was initiated by searching PubMed, EMBASE, Web of Science, and the World Health Organization Global Research Database on COVID-19 (427) weekly from inception through April 2, 2021, with no language restrictions. Our search terms included “SARS-CoV-2,” “novel coronavirus,” “coronavirus 2019,” “COVID-19,” “COVID 2019” AND “asymptomatic,” “no symptoms,” “presymptomatic,” “paucisymptomatic,” “sub-clinical,” “silent transmission,” “silent infection,” “without any symptoms,” and “without symptoms” (*SI Appendix*, Table S1). All studies of any design that included these terms, were published after January 1, 2020, and described the symptom status of COVID-19 cases were considered in the screening step. No changes were made to the search criteria after the study initiation on May 1, 2020. The study protocol is available in the Open Science Framework online public database, registration DOI: 10.17605/OSF.IO/ZCJ62.

All articles were double-screened (by P.S. and C.F.Z.) based on the title and abstract. Studies were excluded if they were 1) duplicate publications, 2) editorials, reviews, discussions, or opinion pieces, 3) ambiguous about the presence of silent infection, 4) modeling studies without primary data, 5) based on fewer than two cases, 6) not conducted in humans, or 7) retracted. All identified full-text articles were reviewed by P.S. and C.F.Z. For each full-text article, we manually searched references for additional relevant studies. Studies included in our meta-analysis either reported laboratory confirmations of COVID-19 at a single time point, providing a snapshot of disease prevalence in the study subjects, or reported longitudinal data over a period of follow-up.

Risk of bias was assessed independently by two authors, and consensus was achieved through discussion. We adapted the ROBINS-I checklist (428) to include seven items: 1) enrollment of all patients satisfying the criteria for inclusion, 2) enrollment of cases regardless of symptom status, 3) confirmation of cases using RT-PCR, 4) symptoms monitored by clinicians rather than self-reporting, 5) symptom assessment at the end of the follow-up period, 6) symptom follow-up duration of at least 7 d, and 7) loss to follow-up less than 5%.

### Data Analysis.

We conducted a meta-analysis using the studies identified through our systematic review to determine the prevalence of those truly asymptomatic among infected individuals. To delineate true asymptomaticity from the combination of asymptomatic and presymptomatic infections, we pursued two complementary analyses: 1) a single-step analysis based on reports of those who were asymptomatic at the end of a follow-up period and 2) a two-step analysis first evaluating the percentage of infections without symptoms at the time of testing and then assessing asymptomaticity by subtracting those that progressed to develop symptoms. In the single-step analysis, we calculated asymptomaticity as the percentage of confirmed COVID-19 cases that continued to exhibit no clinical symptoms for at least 7 d after testing, whether or not symptom status was reported specifically at the time of testing. In the two-step analysis, we focused on a subset of studies that distinguished asymptomatic cases from those that were presymptomatic by reporting symptoms at time of testing as well as conducting follow-up of symptoms for at least 7 d after testing. In both analyses, we removed index case(s) from the denominator of our calculations to minimize representational bias that would result in overestimation of symptomaticity. As a sensitivity analysis, we repeated our calculations including index cases. For studies that did not follow a population screening design, we assumed that single infections without an epidemiological link were necessarily detected due to their symptoms. Therefore, we subset the calculations to include only those infections which were part of a cluster.

To calculate pooled estimates, study outcomes were logit transformed, each study was assigned a weight using the inverse variance method (429), the DerSimonian−Laird estimator was applied to evaluate between-study variance (430), and the Clopper–Pearson method was used to determine CIs (431). Given heterogeneity in asymptomatic percentages estimated across studies, we used a random-effects meta-analysis model, applying the Hartung and Knapp (432) method to adjust test statistics and CIs for the random effect. We evaluated small-study effects visually with a contour-enhanced funnel plot and statistically with Egger’s test (433). As a sensitivity analysis, we excluded studies with a small sample size (<10 infections), and we considered whether their removal impacted the pooling of results.

We conducted subgroup analysis stratified by age class, study design (population screening or not), publication date, duration of symptom follow-up, geographic location, and setting (community, healthcare facility, household, long-term care facilities, and other which encompassed schools, ships, conference, call centers, labor and delivery units, homeless shelters, and detention facilities). For subgroup analysis involving age class, we selected studies where all confirmed cases were either children (0 y to 18 y), adults (19 y to 59 y) or the elderly (≥60 y). We evaluated sex-based differences in asymptomaticity by selecting only those studies that stratified asymptomatic cases with respect to sex. For each of these studies we calculated the IRR, which was the ratio of the asymptomatic percentage in males relative to that in females. A similar analysis was performed to evaluate the asymptomaticity in cases with comorbidity relative to those without.

We next evaluated the impact of sample selection bias arising from higher participation among those experiencing symptoms in studies with voluntary participation. In this analysis, we calculated the pooled asymptomaticity after restricting to a smaller subset of studies that performed screening of every individual at the study setting. To avoid age-dependent bias in asymptomaticity, we removed studies where all participants belonged to a single age class (children, adults, or the elderly). Out of the 25 studies selected, 7 studies performed screening of all close household contacts (64, 80, 83, 103, 117, 131), 3 screened all flight passengers (28, 84, 91), and 2 screened all members of a tourist/pilgrim group (94, 129). Others were based on screening of healthcare workers (25, 110), inpatients admitted for non−COVID-19 reasons (19, 50, 59, 72, 108, 113), rigorously community screening (82, 166), travelers (18, 180), and those associated with a detention facility (92).

The meta-analysis and subgroup analyses were conducted using the metaprop function from the R package meta. Meta-analyses of sex-based and comorbidity-based differences in asymptomaticity were performed using the rma function from the R package metafor.

## Supplementary Material

Supplementary File

## Data Availability

All study data are included in the article and *SI Appendix*.

## Change History

August 27, 2021: Table 1 has been updated.
