# Supplementary material for: Asymptomatic SARS-CoV-2 infection: A systematic review and meta-analysis
Source: Proc Natl Acad Sci U S A. 2021 Aug 10;118(34):e2109229118. doi: 10.1073/pnas.2109229118 (PMC8403749; doi:10.1073/pnas.2109229118)
Supplement: Supplementary File [file pnas.2109229118.sapp.pdf]

PNAS
www.pnas.org

Supplementary Information for

Asymptomatic SARS-CoV-2 infection: a systematic review and meta-analysis.

Authors: Pratha Sah[1], Meagan C. Fitzpatrick[1,2], Charlotte F. Zimmer[1], Elaheh Abdollahi[3], Lyndon Juden-Kelly[3], Seyed M. Moghadas[3], Burton H. Singer[4], Alison P. Galvani[1]

[1]Center for Infectious Disease Modeling and Analysis (CIDMA), Yale School of Public Health, New Haven, CT 06520, USA; [2] Center for Vaccine Development and Global Health, University of Maryland School of Medicine, Baltimore, MD 21201, USA; [3] Agent-Based Modelling Laboratory, York University, Toronto, Ontario M3J 1P3, Canada; [4] Emerging Pathogens Institute, University of Florida, P.O. Box 100009, Gainesville, FL 32610, USA

Corresponding author: Burton H. Singer, Emerging Pathogens Institute, University of Florida, P.O. Box 100009, Gainesville, FL 32610, USA, (352) 273-9572, bhsinger@epi.ufl.edu.

This PDF file includes:

Figures S1 to S3
Tables S1 to S3
SI References

Table S1. Keywords for systematic search.

Database	Search terms
PubMed	("COVID-19"[All Fields] OR "2019-nCov"[All Fields] OR "SARS-CoV-2"[All Fields] OR "novel coronavirus"[All Fields] OR "coronavirus 2019"[All Fields] OR "COVID-2019"[All Fields] OR "COVID 2019"[All Fields]) AND ("asymptomatic"[All Fields] OR "presymptomatic"[All Fields] OR "no symptoms"[All Fields] OR "without any symptoms"[All Fields] OR "without symptoms"[All Fields] OR "paucisymptomatic"[All Fields] OR "sub-clinical"[All Fields] OR "silent transmission"[All Fields] OR "silent infection"[All Fields]")
Embase	(("COVID-19" OR "2019-nCov" OR "SARS-CoV-2" OR "novel coronavirus" OR "coronavirus 2019" OR "COVID-2019" OR "COVID 2019").mp. AND (("asymptomatic" OR "presymptomatic" OR "no symptoms" OR "without any symptoms" OR "without symptoms" OR "paucisymptomatic" OR "sub-clinical" OR "silent transmission" OR "silent infection").mp.)[mp=title, abstract, heading word, drug trade name, original title, device manufacturer, drug manufacturer, device trade name, keyword, floating subheading word, candidate term word]
Web of Science	ts = (("COVID-19" OR "2019-nCov" OR "SARS-CoV-2" OR "novel coronavirus" OR "coronavirus 2019" OR "COVID-2019" OR "COVID 2019") AND ("asymptomatic" OR "presymptomatic" OR "no symptoms" OR "without any symptoms" OR "without symptoms" "paucisymptomatic" OR "sub-clinical" OR "silent transmission" OR "silent infection")
WHO Global Research Database on COVID-19	tw:("asymptomatic" OR "presymptomatic" OR "no symptoms" OR "without any symptoms" OR "without symptoms" OR "paucisymptomatic" OR "sub-clinical" OR "silent transmission" OR "silent infection")

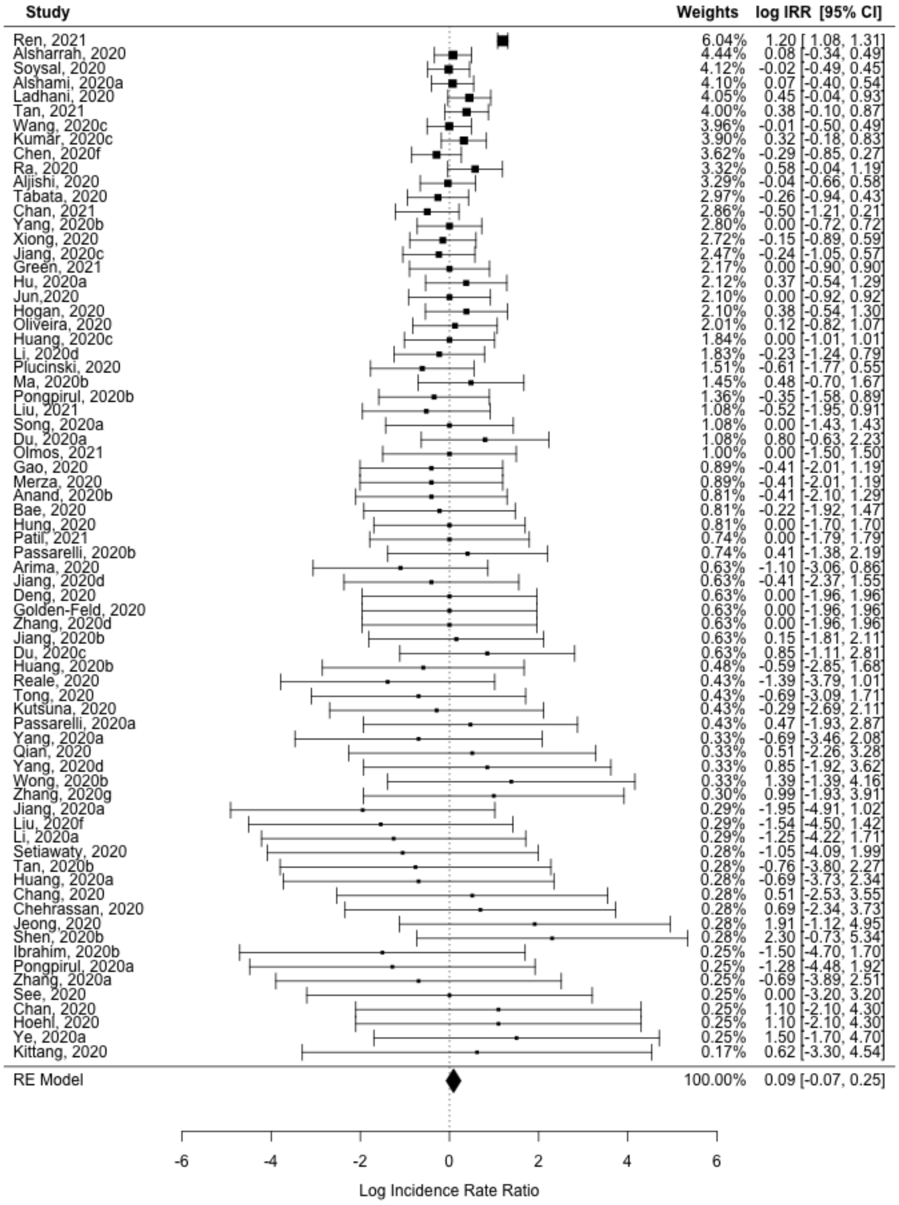

Fig. S1. Meta-analysis of sex differences in asymptomaticity. The size of black squares is proportional to the weight assigned to each study. IRR = Incidence rate ratio of males relative to females, RE=Random effects and CI=Confidence intervals.

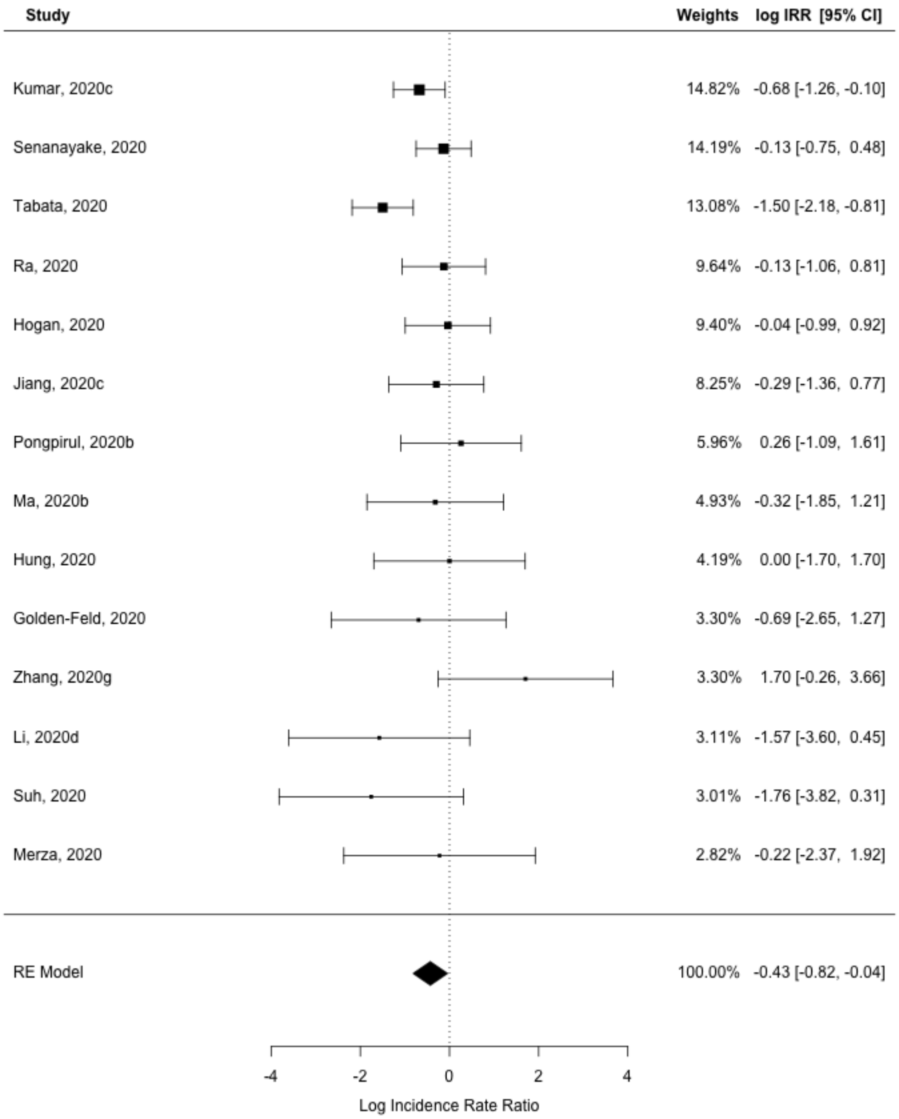

Fig. S2. Meta-analysis of asymptomaticity with respect to comorbidities. The size of black squares is proportional to the weight assigned to each study. IRR = Incidence rate ratio of cases with comorbidities relative to cases that had no comorbidities, RE=Random effects and CI=Confidence intervals.

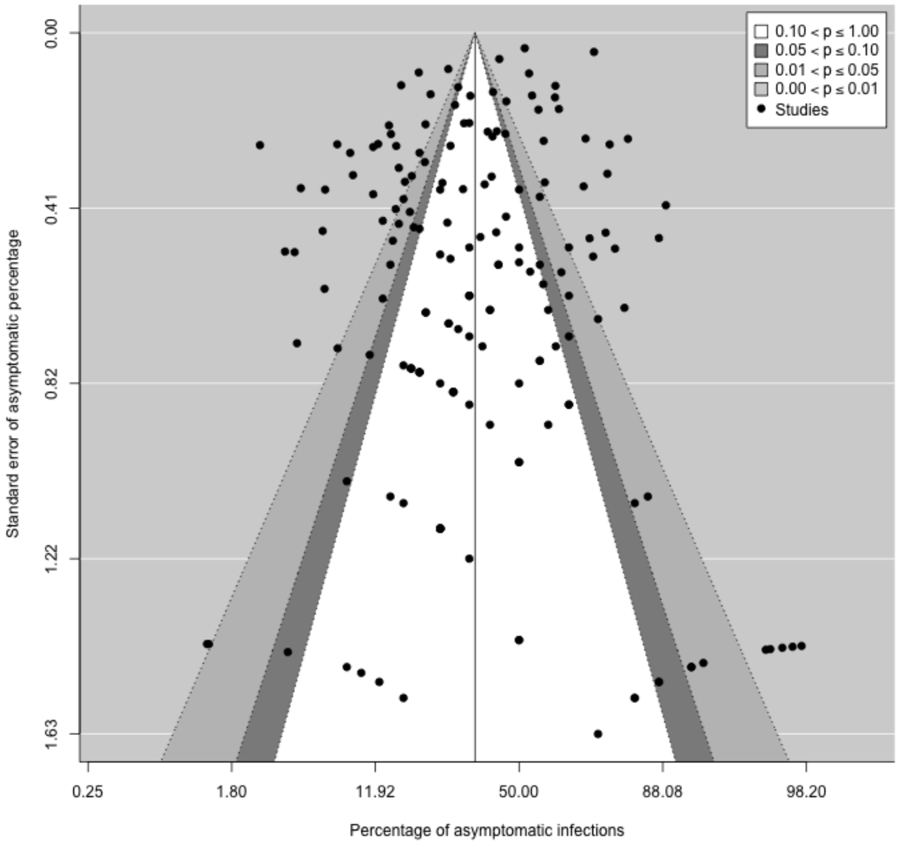

Fig. S3. Contour-enhanced funnel plot to assess small study effects for the asymptomatic percentage. Dots represent individual studies. Background shading indicates the level of statistical significance of the studies. The funnel plot is centered at the asymptomatic percentage of 35.1% (vertical line), the estimate resulting from our primary analysis. Asymmetry in the dot placement within the white region relative to the center line indicates possible areas of publication bias.

Table S2. Characteristics of studies included in the meta-analysis. Index cases are the first symptomatic case within a cluster of infections. Silent infections are those which displayed no symptoms at or prior to the time of testing. Presymptomatic are those which were silent at the time of testing and developed symptoms at a later time point. Asymptomatic are those which were silent at the time of testing and did not develop symptoms within the follow-up period. For categorization of age class, children are 0-18y, adults are 19-59y, and elderly are above 60y. Studies with cross-sectional or screening design were considered to have zero index cases since in these studies there were no sample selection bias arising from higher participation among those experiencing symptoms. For studies with population screening design, 22% reported at least one index case preceding the implementation of COVID-19 screening. NR = not reported; NA = not applicable.

Study	Cases	Index cases	No symptoms	Presymptomatic	Asymptomatic	Publication date	Age class	Study design	Study setting	Country
**Abeysuriya, 2020**[1]	7	0	6	NR	NR	7/20	adults	cross-sectional	other	UK
**Abraha, 2021**[2]	2,617	0	2423	488	1935	03/2021	mixed	population screening	health care facility	Ethiopia
**Adetola, 2020**[3]	9	0	5	NR	NR	10/20	children	prospective cohort	health care facility	Sierra Leone
**Adorni, 2020**[4]	856	0	118	NR	NR	9/20	mixed	cross-sectional	community	Italy
**Aherfi, 2020**[5]	53	6	9	NR	NR	8/20	mixed	prospective cohort	community	France
**Al-Qahtani, 2020**[6]	188	0	144	27	117	11/20	mixed	population screening	community	Bahrain
**Al-Shamsi, 2020b**[7]	32	0	25	19	6	11/20	mixed	population screening	health care facility	UAE
**Albalate, 2020**[8]	37	0	15	NR	NR	4/20	mixed	prospective cohort	health care facility	Spain
**Almadhi, 2021**[9]	1,289	0	1127	NR	NR	02/2021	mixed	contact tracing	community	Bahrain
**Almazeedi, 2020**[10]	1,096	0	508	35	473	7/20	mixed	population screening	community	Kuwait

Study	Cases	Index cases	No symptoms	Presymptomatic	Asymptomatic	Publication date	Age class	Study design	Study setting	Country
**Alsharrah, 2020**[11]	134	23	94	3	91	11/20	children	retrospective cohort	health care facility	Kuwait
**Alvarado, 2020**[12]	736	0	NR	NR	146	10/20	adults	population screening	other	USA
**An, 2020**[13]	25	3	25	9	16	4/20	mixed	retrospective cohort	health care facility	China
**Anand, 2020b**[14]	7	0	6	0	6	9/20	children	population screening	health care facility	India
**Andrikopoulou,**	158	0	63	NR	NR	8/20	adults	prospective cohort	other	USA
**Arima, 2020**[16]	12	0	6	2	4	4/20	adults	population screening	community	Japan
**Arnold, 2021**[17]	103	0	38	NR	NR	1/21	mixed	population screening	health care facility	USA
**Arons, 2020**[18]	48	0	27	24	3	5/20	elderly	population screening	nursing facility	USA
**Ashahrani, 2020**[19]	18	0	12	0	12	12/20	mixed	population screening	health care facility	Saudi Arabia
**Aslam, 2020**[20]	65	0	41	3	38	10/20	NR	population screening	health care facility	USA
**Atakla, 2020**[21]	36	23	11	NR	NR	12/2020	children	retrospective cohort	health care facility	Guinea
**Atalla, 2020**[22]	111	0	47	37	10	12/20	elderly	population screening	nursing facility	USA
**Bae, 2020**[23]	7	0	7	1	6	8/20	mixed	population screening	community	South Korea
**Baggett, 2020**[24]	147	0	129	NR	NR	4/20	mixed	population screening	other	USA

Study	Cases	Index cases	No symptoms	Presymptomatic	Asymptomatic	Publication date	Age class	Study design	Study setting	Country
**Bai, 2020a**[25]	6	1	1	NR	NR	2/20	mixed	prospective cohort	household	China
**Bai, 2020b**[26]	7	1	5	NR	NR	2/20	mixed	population screening	household	China
**Bayle, 2020**[27]	32	0	24	NR	NR	12/20	NA	population screening	other	France
**Beiting, 2021**[28]	172	0	69	19	50	03/2021	elderly	population screening	nursing facility	USA
**Bender, 2020**[29]	8	0	8	3	5	9/20	adults	population screening	other	USA
**Bender, 2021**[30]	53	0	7	NR	NR	02/2021	mixed	contact tracing	community	Germany
**Berghoff, 2020**[31]	4	0	2	NR	NR	8/20	mixed	prospective cohort	health care facility	Austria
**Berry, 2021**[32]	91	0	56	NR	NR	02/2021	mixed (children and adults)	cross-sectional	other	USA
**Bi, 2020**[33]	87	0	17	NR	NR	4/20	mixed	cross-sectional	community	China
**Bigelow, 2020**[34]	507	153	281	NR	NR	7/20	NA	population screening	nursing facility	USA
**Blain, 2020a**[35]	38	0	NR	NR	6	6/20	elderly	population screening	nursing facility	USA
**Blain, 2020b**[36]	38	0	3	3	0	10/20	elderly	population screening	nursing facility	France
**Blain, 2021**[37]	161	0	28	14	14	02/2021	elderly	population screening	nursing facility	France
**Blitz, 2020**[38]	64	0	45	NR	NR	8/20	adults	population screening	other	USA

Study	Cases	Index cases	No symptoms	Presymptomatic	Asymptomatic	Publication date	Age class	Study design	Study setting	Country
**Blumberg, 2020**[39]	7	0	6	NR	NR	7/20	children	retrospective cohort	health care facility	USA
**Brandstetter, 2020**[40]	31	1	1	NR	NR	5/20	mixed	population screening	health care facility	Germany
**Cabraal, 2021**[41]	78	26	43	NR	NR	03/2021	children	retrospective cohort	health care facility	Sri Lanka
**Camara, 2020**[42]	189	0	99	NR	NR	12/20	children	cross-sectional	health care facility	Guinea
**Campbell, 2020**[43]	30	0	22	NR	NR	5/20	adults	population screening	other	USA
**Cardona-Hernandez, 2020**[44]	11	2	6	NR	NR	11/20	children	prospective cohort	community	Italy
**Carroll, 2020**[45]	310	0	209	NR	NR	10/20	mixed	cross-sectional	community	Ireland
**Cattelan, 2020**[46]	395	0	109	NR	NR	8/20	mixed	population screening	health care facility	Italy
**Cesilia, 2021**[47]	6	1	4	NR	NR	01/2021	mixed (children and adults)	contact tracing	household	Malaysia
**Chan, 2020**[48]	6	2	1	0	1	1/20	mixed	population screening	household	China
**Chan, 2021**[49]	568	0	58	43	15	03/2021	mixed (adults and elderly)	retrospective cohort	other	USA
**Chang, 2020**[50]	217	0	75	NR	NR	5/20	mixed	cross-sectional	community	South Korea
**Chau, 2020**[51]	14	1	7	0	7	6/20	adults	prospective cohort	health care facility	Vietnam

**Study**	Cases	Index cases	No symptoms	Presymptomatic	Asymptomatic	Publication date	Age class	Study design	Study setting	Country
**Chehrassan, 2020**[52]	7	0	6	3	2	4/20	mixed	population screening	health care facility	Iran
**Chekhlabi, 2020**[53]	15	0	5	NR	NR	4/20	children	retrospective cohort	health care facility	Morocco
**Chen, 2020**[54]	141	51	32	NR	NR	6/20	mixed	retrospective cohort	health care facility	China
**Chen, 2020a**[55]	90	0	29	NR	NR	4/20	adults	retrospective cohort	health care facility	China
**Chen, 2020c**[56]	132	0	22	NR	NR	5/20	NR	cross-sectional	community	China
**Chen, 2020d**[57]	16	0	6	NR	NR	7/20	adults	population screening	flight	China
**Cheng, 2020**[58]	22	0	4	NR	NR	5/20	mixed	prospective cohort	community	Taiwan
**Chiu, 2020**[59]	4	0	1	NR	NR	4/20	mixed	prospective cohort	household	Taiwan
**Choudhury, 2020**[60]	6	0	1	0	1	10/20	adults	prospective cohort	health care facility	India
**Clarke, 2020**[61]	129	0	52	NR	NR	9/20	mixed	prospective cohort	health care facility	UK
**Coppeta, 2020**[62]	12	0	1	1	0	12/20	NR	population screening	health care facility	Italy
**Corcorann, 2020**[63]	10	2	4	1	3	8/20	elderly	prospective cohort	health care facility	USA
**Cosma, 2020**[64]	14	0	6	0	6	7/20	adults	population screening	other	Italy
**Crameri, 2020**[65]	145	0	77	NR	NR	9/20	adults	prospective cohort	other	Switzerland

**Study**	Cases	Index cases	No symptoms	Presymptomatic	Asymptomatic	Publication date	Age class	Study design	Study setting	Country
**Cruz-Lemini, 2021**[66]	279	0	NR	NR	174	1/21	adults	population screening	other	Spain
**D'Ambrosi, 2020**[67]	6	0	2	NR	NR	10/20	adults	population screening	other	Italy
**da Cunha, 2020**[68]	38	0	5	NR	NR	7/20	elderly	retrospective cohort	health care facility	Spain
**da Silva, 2020**[69]	5	1	1	NR	NR	7/20	mixed	retrospective cohort	household	Brazil
**Danis, 2020**[70]	13	1	1	0	1	4/20	mixed	prospective cohort	community	France
**Dbeido, 2021**[71]	46	0	17	NR	NR	02/2021	mixed	cross-sectional	health care facility	USA
**de Jesus, 2020**[72]	7	1	2	NR	NR	8/20	mixed	prospective cohort	household	Brazil
**Dhuyvetter, 2020**[73]	23	0	10	NR	NR	10/20	mixed	population screening	other	USA
**Diaz-Corvillon,**	37	0	16	NR	NR	9/20	mixed	population screening	other	Chile
**Dixon, 2021**[75]	368	0	91	NR	NR	03/2021	mixed	cross-sectional	community	USA
**Donahue, 2020**[76]	241	0	78	NR	NR	8/20	mixed	cross-sectional	other	USA
**Dora, 2020**[77]	19	1	14	8	6	5/20	elderly	population screening	nursing facility	USA
**Du, 2020a**[78]	14	0	NR	NR	8	4/20	children	retrospective cohort	health care facility	China
**Du, 2020b**[79]	182	4	55	NR	NR	6/20	children	retrospective cohort	health care facility	China

Study	Cases	Index cases	No symptoms	Presymptomatic	Asymptomatic	Publication date	Age class	Study design	Study setting	Country
**Du, 2020c**[80]	10	0	5	1	4	5/20	children	retrospective cohort	health care facility	China
**Duan, 2020**[81]	31	3	4	NR	NR	4/20	children	retrospective cohort	health care facility	China
**Edlow, 2020**[82]	64	0	23	NR	NR	12/20	adults	prospective cohort	other	USA
**El-Sokkary, 2021**[83]	77	0	7	NR	NR	1/21	NA	cross-sectional	health care facility	Egypt
**Eythorsson, 2020**[84]	178	0	38	13	25	12/20	mixed	population screening	community	Iceland
**Fakhim, 2021**[85]	21	0	14	NR	NR	01/2021	NA	cross-sectional	health care facility	Iran
**Felice, 2020**[86]	18	0	6	NR	NR	5/20	mixed	cross-sectional	health care facility	Italy
**Fusco, 2020**[87]	4	0	2	1	1	6/20	mixed	population screening	health care facility	Italy
**Gao, 2020**[88]	15	1	NR	NR	6	4/20	mixed	retrospective cohort	community	China
**Gaur, 2020**[89]	26	1	8	NR	NR	7/20	mixed	retrospective cohort	health care facility	India
**Golden-Feld, 2020**[90]	6	0	4	0	4	11/20	elderly	retrospective cohort	other	Israel
**Goldfarb, 2020**[91]	20	0	9	NR	NR	5/20	adults	prospective cohort	other	USA
**Gonfiotti, 2020**[92]	5	1	1	0	1	8/20	elderly	prospective cohort	health care facility	Italy
**Gong, 2020**[93]	14	3	2	NR	NR	8/20	mixed	prospective cohort	community	China

**Study**	Cases	Index cases	No symptoms	Presymptomatic	Asymptomatic	Publication date	Age class	Study design	Study setting	Country
**Graham, 2020**[94]	126	0	54	NR	NR	6/20	elderly	prospective cohort	nursing facility	UK
**Grechukhina, 2020**[95]	141	0	44	NR	NR	10/20	adults	prospective cohort	other	USA
**Green, 2021**[96]	22	0	22	0	22	1/21	mixed	population screening	nursing facility	UK
**Greiner, 2020**[97]	9	0	1	NR	NR	12/20	adults	prospective cohort	health care facility	Germany
**Grijalva, 2020**[98]	102	0	61	27	34	10/20	mixed	population screening	household	USA
**Gruskay, 2020**[99]	16	0	9	NR	NR	6/20	mixed	population screening	health care facility	USA
**Gudbjartsson,**	100	0	31	NR	NR	6/20	mixed	cross-sectional	community	Iceland
**Guo, 2020**[101]	341	131	20	NR	NR	8/20	children	retrospective cohort	health care facility	China
**Gupta, 2020b**[102]	19	0	19	0	19	12/20	children	population screening	health care facility	India
**Gupta, 2021**[103]	127	0	75	3	72	02/2021	NA	population screening	community	India
**Gutman, 2021**[104]	7	0	NR	NR	4	03/2021	adults	population screening	health care facility	USA
**Han, 2020**[105]	91	19	38	18	20	8/20	children	retrospective cohort	health care facility	South Korea
**Han, 2020b**[106]	10	3	2	NR	NR	7/20	mixed	prospective cohort	other	South Korea
**Han, 2020c**[107]	311	70	49	NR	NR	10/20	mixed	prospective cohort	community	China

**Study**	Cases	Index cases	No symptoms	Presymptomatic	Asymptomatic	Publication date	Age class	Study design	Study setting	Country
**Handal, 2021**[108]	2	0	1	1	0	02/2021	NA	population screening	health care facility	Norway
**Harada, 2020**[109]	73	0	38	5	33	10/20	mixed	population screening	health care facility	Japan
**Hasanoglu, 2020**[110]	60	45	15	0	15	11/20	mixed	prospective cohort	health care facility	Turkey
**Hcini, 2020**[111]	137	0	103	16	87	11/20	NR	population screening	other	French Guiana
**He, 2020a**[112]	94	48	2	0	2	4/20	adults	retrospective cohort	health care facility	China
**He, 2020c**[113]	35	7	7	NR	NR	12/20	children	retrospective cohort	health care facility	China
**Hempel, 2020**[114]	78	0	75	NR	NR	10/20	mixed	cross-sectional	health care facility	Germany
**Herraiz, 2020**[115]	2	0	1	NR	NR	7/20	adults	retrospective cohort	other	Spain
**Hijnen, 2020**[116]	12	1	2	NR	NR	5/20	adults	prospective cohort	other	Germany
**Hoehl, 2020**[117]	2	0	2	1	1	3/20	mixed	population screening	community	Germany
**Hogan, 2020**[118]	26	0	26	6	20	11/20	NR	population screening	health care facility	USA
**Hong, 2020**[119]	41	25	15	NR	NR	11/20	mixed	retrospective cohort	health care facility	China
**Hu, 2020a**[120]	24	0	24	5	19	3/20	mixed	prospective cohort	community	China
**Hu, 2020b**[121]	7	0	1	NR	NR	7/20	adults	prospective cohort	other	China

**Study**	Cases	Index cases	No symptoms	Presymptomatic	Asymptomatic	Publication date	Age class	Study design	Study setting	Country
**Hu, 2021**[122]	471	0	104	NR	NR	03/2021	mixed	contact tracing	community	China
**Hua, 2020**[123]	43	3	10	0	10	6/20	children	retrospective cohort	health care facility	China
**Huang, 2020a**[124]	7	1	4	2	2	4/20	mixed	retrospective cohort	household	China
**Huang, 2020b**[125]	8	1	4	0	4	6/20	mixed	retrospective cohort	household	China
**Huang, 2020c**[126]	16	0	16	0	16	9/20	mixed	population screening	health care facility	China
**Huete-Perez,**	122	0	67	NR	NR	1/21	mixed	population screening	health care facility	Nicaragua
**Hung, 2020**[128]	9	0	7	1	6	6/20	mixed	population screening	other	Hong Kong
**Ibrahim, 2020b**[129]	5	1	1	0	1	5/20	children	retrospective cohort	health care facility	Nigeria
**Ing, 2020**[130]	128	1	104	NR	NR	5/20	mixed	prospective cohort	other	Argentina
**Inui, 2020**[131]	104	0	76	NR	NR	3/20	mixed	retrospective cohort	other	Japan
**Isoldi, 2021**[132]	15	0	4	NR	NR	02/2021	children	population screening	health care facility	Italy
**Jani, 2021**[133]	34	1	28	NR	NR	02/2021	NA	population screening	other	USA
**Jatt, 2020**[134]	78	2	22	NR	NR	6/20	mixed	population screening	health care facility	USA
**Jeong, 2020**[135]	40	13	7	5	2	9/20	mixed	retrospective cohort	health care facility	South Korea

**Study**	Cases	Index cases	No symptoms	Presymptomatic	Asymptomatic	Publication date	Age class	Study design	Study setting	Country
**Jiang, 2020a**[136]	8	2	5	2	3	4/20	mixed	prospective cohort	household	China
**Jiang, 2020b**[137]	13	1	4	0	4	4/20	mixed	population screening	household	China
**Jiang, 2020d**[138]	10	0	4	0	4	10/20	children	retrospective cohort	health care facility	China
**Jones, 2020**[139]	20	0	7	2	5	6/20	mixed	population screening	health care facility	UK
**Jones, 2021**[140]	87	0	24	NR	NR	1/21	NA	population screening	community	USA
**Joshi, 2020**[141]	25	0	25	0	25	10/20	NR	population screening	health care facility	India
**Jui-Yao, 2020**[142]	55	23	9	NR	NR	12/20	mixed	retrospective cohort	community	Taiwan
**Jung, 2020**[143]	3	1	1	0	1	6/20	mixed	prospective cohort	health care facility	South Korea
**Just, 2020**[144]	40	15	14	NR	NR	12/20	mixed	retrospective cohort	health care facility	Germany
**Kamiya, 2020**[145]	544	1	314	NR	NR	8/20	mixed	prospective cohort	other	Japan
**Kang, 2020**[146]	7	0	2	0	2	12/20	children	retrospective cohort	health care facility	China
**Karout, 2020**[147]	76	0	76	64	12	9/20	mixed	population screening	community	USA
**Kassem, 2020**[148]	16	0	10	NR	NR	7/20	adults	prospective cohort	health care facility	Egypt
**Ke, 2020**[149]	25	7	8	NR	NR	7/20	children	prospective cohort	community	China

Study	Cases	Index cases	No symptoms	Presymptomatic	Asymptomatic	Publication date	Age class	Study design	Study setting	Country
**Kenelly, 2020**[150]	710	0	NR	NR	193	9/20	NR	population screening	nursing facility	Ireland
**Khalil, 2020**[151]	9	0	8	NR	NR	5/20	adults	prospective cohort	other	UK
**Khondaker, 2020**[152]	26	0	7	NR	NR	12/20	children	population screening	health care facility	Bangladesh
**Ki, 2020**[153]	28	16	3	NR	NR	2/20	mixed	retrospective cohort	health care facility	South Korea
**Kimball, 2020**[154]	23	0	13	10	3	4/20	elderly	population screening	nursing facility	USA
**Kirshblum, 2020**[155]	7	0	7	5	2	7/20	mixed	population screening	health care facility	USA
**Kittang, 2020**[156]	40	3	9	9	0	6/20	elderly	retrospective cohort	nursing facility	Norway
**Koizumi, 2020**[157]	103	74	21	NR	NR	6/20	mixed	retrospective cohort	other	Japan
**Kong, 2020a**[158]	28	18	3	NR	NR	2/20	mixed	retrospective cohort	community	South Korea
**Kristiansen, 2021**[159]	187	75	21	NR	NR	1/21	mixed	population screening	community	Denmark
**Krone, 2021**[160]	80	1	31	0	31	1/21	elderly	population screening	nursing facility	Germany
**Kruger, 2021**[161]	27	0	11	4	7	1/21	mixed	population screening	health care facility	Germany
**Kute, 2020**[162]	250	139	15	NR	NR	12/20	mixed	retrospective cohort	health care facility	India
**Kute, 2021**[163]	75	52	17	NR	NR	03/2021	NA	retrospective cohort	health care facility	India

**Study**	Cases	Index cases	No symptoms	Presymptomatic	Asymptomatic	Publication date	Age class	Study design	Study setting	Country
**Kutsuna, 2020**[164]	11	0	6	3	3	6/20	mixed	population screening	community	Japan
**LaCourse, 2020**[165]	5	0	1	NR	NR	5/20	adults	population screening	other	USA
**Ladhani, 2020**[166]	158	0	97	25	72	9/20	mixed	population screening	nursing facility	UK
**Lai, 2020**[167]	110	0	3	NR	NR	5/20	adults	prospective cohort	health care facility	China
**Lamichhane,**	9	1	5	3	2	03/2021	mixed (children and adults)	contact tracing	household	Canada
**Lavezzo, 2020**[169]	81	0	34	NR	NR	6/20	mixed	cross-sectional	community	Italy
**Le, 2020**[170]	12	6	1	NR	NR	4/20	mixed	prospective cohort	community	Vietnam
**Lee, 2020b**[171]	694	201	NR	NR	80	7/20	mixed	retrospective cohort	health care facility	South Korea
**Letizia, 2020**[172]	51	0	46	NR	NR	12/20	adults	prospective cohort	other	USA
**Lewis, 2020b**[173]	6	0	3	NR	NR	7/20	mixed	cross-sectional	health care facility	USA
**Li, 2020a**[174]	18	4	9	6	3	7/20	mixed	prospective cohort	health care facility	China
**Li, 2020b**[175]	252	114	74	NR	NR	6/20	mixed	retrospective cohort	health care facility	China
**Li, 2020c**[176]	7	0	6	NR	NR	7/20	mixed	retrospective cohort	health care facility	China
**Li, 2020d**[177]	39	2	19	4	15	9/20	children	prospective cohort	health care facility	Singapore

**Study**	Cases	Index cases	No symptoms	Presymptomatic	Asymptomatic	Publication date	Age class	Study design	Study setting	Country
**Li, 2020e**[178]	52	36	9	NR	NR	12/20	mixed	retrospective cohort	health care facility	China
**Liao, 2020a**[179]	46	35	4	2	2	5/20	mixed	retrospective cohort	health care facility	China
**Lin, 2020**[180]	161	122	6	NR	NR	10/20	mixed	retrospective cohort	health care facility	China
**Liu, 2020b**[181]	45,561	0	45,561	NR	NR	2/20	mixed	cross-sectional	community	China
**Liu, 2020c**[182]	321	0	11	NR	NR	5/20	mixed	prospective cohort	community	Taiwan
**Liu, 2020d**[183]	24	12	3	NR	NR	5/20	mixed	retrospective cohort	health care facility	China
**Liu, 2020e**[184]	625	310	24	NR	NR	8/20	mixed	retrospective cohort	health care facility	China
**Liu, 2020f**[185]	5	2	4	1	3	7/20	mixed	population screening	household	China
**Liu, 2020g**[186]	77	1	21	NR	NR	6/20	mixed	prospective cohort	community	China
**Liu, 2020j**[187]	41	11	11	NR	NR	12/20	mixed	retrospective cohort	community	China
**Liu, 2021**[188]	209	140	16	8	8	03/2021	mixed	retrospective cohort	health care facility	China
**Lokken, 2020**[189]	46	0	3	NR	NR	5/20	adults	retrospective cohort	other	USA
**Lombardi, 2020**[190]	139	1	28	11	17	6/20	mixed	population screening	health care facility	Italy
**London, 2020**[191]	68	0	22	NR	NR	8/20	adults	retrospective cohort	other	USA

**Study**	Cases	Index cases	No symptoms	Presymptomatic	Asymptomatic	Publication date	Age class	Study design	Study setting	Country
**Lopez, 2020**[192]	7	0	4	NR	NR	9/20	children	retrospective cohort	other	USA
**Lu, 2020a**[193]	171	17	27	NR	NR	3/20	children	retrospective cohort	health care facility	China
**Lu, 2020b**[194]	619	0	318	NR	NR	2/20	mixed	prospective cohort	other	Japan
**Luo, 2020a**[195]	127	0	NR	NR	8	8/20	mixed	population screening	community	China
**Luo, 2020b**[196]	6	1	5	0	5	4/20	mixed	population screening	household	China
**Ly, 2020**[197]	226	37	46	NR	NR	12/20	mixed	retrospective cohort	nursing facility	France
**Lytras, 2020**[198]	40	0	39	4	35	4/20	mixed	population screening	community	Greece
**Ma, 2020b**[199]	47	12	NR	NR	11	5/20	mixed	retrospective cohort	health care facility	China
**Macartney, 2020**[200]	18	0	NR	NR	5	8/20	NR	prospective cohort	other	Australia
**Maechler, 2020**[201]	333	0	12	NR	NR	8/20	mixed	cross-sectional	community	Germany
**Maggiolo, 2020**[202]	55	15	10	NR	NR	7/20	adults	prospective cohort	health care facility	Italy
**Malagon-Rojas, 2020**[203]	16	0	NR	NR	13	09/2020	NA	population screening	other	Colombia
**Malheiro, 2020**[204]	98	0	12	NR	NR	9/20	mixed	retrospective cohort	community	Portugal
**Maltezou, 2020a**[205]	68	23	16	NR	NR	8/20	mixed	prospective cohort	household	Greece

**Study**	Cases	Index cases	No symptoms	Presymptomatic	Asymptomatic	Publication date	Age class	Study design	Study setting	Country
**Maltezou, 2020b**[206]	203	67	111	NR	NR	10/20	children	prospective cohort	community	Greece
**Mao, 2020**[207]	226	67	41	NR	NR	10/20	mixed	Contact tracing	community	China
**Marcus, 2021**[208]	20	8	6	NR	NR	1/21	mixed	retrospective cohort	health care facility	Israel
**Marcus, 2021b**[209]	403	0	199	NR	NR	02/2021	NA	cross-sectional	other	USA
**Marks, 2021**[210]	449	0	421	181	240	02/2021	NA	contact tracing	community	Spain
**Marossy, 2020**[211]	146	0	115	NR	NR	9/20	mixed	cross-sectional	nursing facility	UK
**Martin, 2020**[212]	41	0	31	NR	NR	6/20	adults	cross-sectional	health care facility	Belgium
**Martinez-Fierro,**	34	0	5	0	5	10/20	adults	population screening	community	Mexico
**Martini, 2020**[214]	32	0	11	NR	NR	12/20	NR	retrospective cohort	health care facility	Italy
**Massarotti, 2020**[215]	7	0	6	NR	NR	8/20	adults	cross-sectional	other	Italy
**Matin-Villares , 2021**[216]	157	0	11	NR	NR	1/21	NA	prospective cohort	health care facility	Spain
**Meena, 2020**[217]	8	1	4	NR	NR	9/20	NR	prospective cohort	health care facility	India
**Melgosa, 2020**[218]	16	0	3	NR	NR	5/20	children	retrospective cohort	health care facility	Spain
**Menachemi, 2020**[219]	3,605	0	1593	NR	NR	7/20	mixed	cross-sectional	community	USA

Study	Cases	Index cases	No symptoms	Presymptomatic	Asymptomatic	Publication date	Age class	Study design	Study setting	Country
**Menting, 2020**[220]	11	0	2	NR	NR	10/20	mixed	prospective cohort	health care facility	Germany
**Merza, 2020**[221]	15	5	NR	NR	6	5/20	mixed	prospective cohort	health care facility	Iraq
**Meyers, 2020**[222]	81	0	81	23	58	6/20	mixed	population screening	community	USA
**Meyers, 2021**[223]	86	0	86	19	67	1/21	mixed	prospective cohort	community	USA
**Migisha, 2020**[224]	54	20	25	5	20	11/20	mixed	prospective cohort	community	Uganda
**Miller, 2020**[225]	23	0	10	NR	NR	5/20	adults	prospective cohort	other	USA
**Min, 2021**[226]	74	0	17	NR	NR	1/21	mixed	population screening	health care facility	China
**Miyahara, 2021**[227]	453	306	67	NR	NR	02/2021	mixed	prospective cohort	household	Japan
**Mostafa, 2020**[228]	170	0	116	NR	NR	10/20	mixed	cross-sectional	health care facility	Egypt
**Myles, 2020**[229]	8	0	4	NR	NR	11/2020	NA	cross-sectional	health care facility	Australia
**Nakajo, 2021**[230]	36	1	12	NR	NR	02/2021	NA	contact tracing	community	Japan
**Namal, 2020**[231]	28	0	25	NR	NR	12/20	mixed	prospective cohort	health care facility	Turkey
**Negro, 2021**[232]	118	0	78	NR	NR	01/2021	NA	cross-sectional	health care facility	Spain
**Ng, 2020a**[233]	3	0	1	1	0	3/20	mixed	population screening	community	China

Study	Cases	Index cases	No symptoms	Presymptomatic	Asymptomatic	Publication date	Age class	Study design	Study setting	Country
**Nicolas, 2020**[234]	590	293	134	45	89	12/2020	NA	prospective cohort	health care facility	Spain
**Nishiura, 2020**[235]	8	0	5	NR	NR	2/20	NR	prospective cohort	community	Japan
**Njuguna, 2020**[236]	71	0	32	3	29	7/20	mixed	population screening	other	USA
**Norsa, 2020**[237]	19	0	11	NR	NR	8/20	mixed	cross-sectional	health care facility	Italy
**Oduro-Mensah , 2020**[238]	275	107	142	NR	NR	12/2020	mixed	retrospective cohort	health care facility	Ghana
**Olmos, 2021**[239]	14	0	14	7	7	1/21	NA	population screening	health care facility	Chile
**Ornahgi, 2020**[240]	51	0	13	NR	NR	9/20	adults	prospective cohort	other	Italy
**Ossareh, 2020**[241]	27	0	21	NR	NR	9/20	mixed	prospective cohort	health care facility	Iran
**Oster, 2020**[242]	43	0	5	NR	NR	8/20	NR	cross-sectional	community	Israel
**Panagiotakopoulos,**	105	43	50	NR	NR	9/20	mixed	retrospective cohort	other	USA
**Park, 2020a**[244]	97	2	8	4	4	4/20	mixed	population screening	other	South Korea
**Park, 2020b**[245]	30	2	8	4	4	12/20	mixed	population screening	community	South Korea
**Parri, 2020a**[246]	130	67	17	NR	NR	6/20	children	retrospective cohort	health care facility	Italy
**Parri, 2020b**[247]	170	100	29	NR	NR	12/20	children	retrospective cohort	health care facility	Italy

Study	Cases	Index cases	No symptoms	Presymptomatic	Asymptomatic	Publication date	Age class	Study design	Study setting	Country
**Passarelli, 2020a**[248]	9	0	9	6	3	8/20	mixed	population screening	health care facility	Brazil
**Passarelli, 2020b**[249]	6	0	6	1	5	10/20	mixed	population screening	health care facility	Brazil
**Patberg, 2020**[250]	77	0	67	NR	NR	10/20	NR	retrospective cohort	other	USA
**Patel, 2020a**[251]	35	1	14	1	13	6/20	elderly	population screening	nursing facility	USA
**Patel, 2020b**[252]	2	0	1	NR	NR	8/20	children	population screening	health care facility	USA
**Patil, 2021**[253]	5	0	5	0	5	01/2021	children	population screening	health care facility	USA
**Pavli, 2020a**[254]	48	2	NR	NR	7	6/20	mixed	prospective cohort	community	Greece
**Pavli, 2020b**[255]	5	0	2	NR	NR	9/20	mixed	prospective cohort	community	Greece
**Payne, 2020**[256]	238	0	44	NR	NR	6/20	adults	prospective cohort	other	USA
**Petersen, 2020**[257]	115	0	88	NR	NR	10/20	NR	cross-sectional	community	UK
**Pham, 2020**[258]	270	53	120	31	89	8/20	mixed	prospective cohort	community	Vietnam
**Pirnay, 2020**[259]	9	0	4	NR	NR	8/20	adults	cross-sectional	other	Belgium
**Plucinski, 2020**[260]	66	0	NR	NR	14	8/20	mixed	population screening	other	USA
**Pollan, 2020**[261]	3,054	0	992	NR	NR	7/20	mixed	cross-sectional	community	Spain

Study	Cases	Index cases	No symptoms	Presymptomatic	Asymptomatic	Publication date	Age class	Study design	Study setting	Country
**Pongpirul, 2020a**[262]	11	4	1	0	1	4/20	mixed	prospective cohort	health care facility	Thailand
**Prabhu, 2020**[263]	70	0	42	NR	NR		adults	prospective cohort	other	USA
**Pray, 2020**[264]	78	1	1	NR	NR	10/20	mixed	prospective cohort	other	USA
**Puylaert, 2020**[265]	18	0	NR	NR	2	7/20	mixed	population screening	health care facility	Netherlands
**Qian, 2020**[266]	8	1	2	0	2	3/20	mixed	population screening	household	China
**Qiang, 2020b**[267]	52	15	5	NR	NR	5/20	mixed	prospective cohort	household	China
**Qin, 2020**[268]	80	20	15	4	11	7/20	mixed	retrospective cohort	community	China
**Qiu, 2020a**[269]	36	4	10	NR	NR	3/20	children	retrospective cohort	health care facility	China
**Qiu, 2020b**[270]	104	22	NR	NR	5	5/20	mixed	prospective cohort	health care facility	China
**Rajme-Lopez , 2021**[271]	111	0	31	14	17	02/2021	mixed (adults and elderly)	population screening	health care facility	Mexico
**Ralli, 2020**[272]	12	0	9	0	9	12/20	NR	population screening	other	Italy
**Randremanana , 2021**[273]	5,553	0	3311	NR	NR	02/2021	mixed	cross-sectional	community	Madagascar
**Reale, 2020**[274]	18	7	11	8	3	8/20	mixed	retrospective cohort	health care facility	Italy
**Reale, 2021**[275]	93	0	80	NR	NR	1/21	NA	population screening	other	USA

Study	Cases	Index cases	No symptoms	Presymptomatic	Asymptomatic	Publication date	Age class	Study design	Study setting	Country
**Reid, 2020**[276]	5	0	3	3	0	12/20	NA	population screening	health care facility	Canada
**Ren, 2021**[277]	30	0	NR	NR	6	05/2020	mixed (adults and elderly)	population screening	health care facility	UK
**Rincon, 2020**[278]	36	22	14	5	9	7/20	mixed	population screening	health care facility	Spain
**Rivera, 2020**[279]	61	0	61	7	54	8/20	mixed	population screening	health care facility	USA
**Rivett, 2020**[280]	30	0	NR	NR	6	5/20	mixed	population screening	health care facility	UK
**Roberts, 2021**[281]	30	0	20	5	15	1/21	NA	population screening	health care facility	USA
**Rogers, 2020**[282]	29	0	21	NR	NR	9/20	mixed	cross-sectional	community	USA
**Roxby, 2020**[283]	6	0	4	NR	NR	5/20	elderly	prospective cohort	nursing facility	USA
**Sabetian, 2021**[284]	273	0	97	NR	NR	03/2021	NA	cross-sectional	health care facility	Iran
**Sacco, 2020**[285]	41	3	3	NR	NR	6/20	elderly	retrospective cohort	nursing facility	France
**Saeed, 2020**[286]	28	0	16	0	16	12/20	NR	population screening	health care facility	UK
**Saenz, 2020**[287]	29	0	24	NR	NR	6/20	mixed	prospective cohort	other	Peru
**Saidy, 2020**[288]	3	0	3	0	3	6/20	mixed	population screening	health care facility	Germany
**Sakowicz, 2020**[289]	101	0	24	NR	NR	8/20	adults	retrospective cohort	other	USA

Study	Cases	Index cases	No symptoms	Presymptomatic	Asymptomatic	Publication date	Age class	Study design	Study setting	Country
**Saluja, 2020**[290]	406	140	244	NR	NR	5/20	mixed	retrospective cohort	health care facility	India
**Samrah, 2020**[291]	81	2	37	NR	NR	7/20	mixed	retrospective cohort	health care facility	Jordan
**Sanchez, 2020**[292]	1,207	0	461	NR	NR	7/20	elderly	prospective cohort	nursing facility	USA
**Sastry, 2020**[293]	79	0	12	NR	NR	7/20	mixed	cross-sectional	health care facility	USA
**Saurabh, 2020**[294]	48	0	44	NR	NR	10/20	mixed	prospective cohort	community	India
**Saviron-Cornudella,**	6	0	4	NR	NR	11/20	adults	population screening	other	Spain
**Saviron-Cornudella, 2021**[296]	22	0	20	NR	NR	02/2021	NA	cross-sectional	other	Spain
**Scheier, 2021**[297]	68	0	8	NR	NR	1/21	mixed	cross-sectional	health care facility	Switzerland
**Schwierzeck,**	12	1	6	4	2	4/20	mixed	population screening	health care facility	Germany
**See, 2020**[299]	4	1	2	1	1	3/20	children	prospective cohort	health care facility	Malaysia
**Senanayake,**	53	0	41	0	41	10/20	mixed	population screening	health care facility	Sri Lanka
**Setiawaty, 2020**[301]	11	0	2	0	2	10/20	mixed	prospective cohort	community	Indonesia
**Sharma, 2020c**[302]	121	7	87	NR	NR	12/20	children	prospective cohort	health care facility	Nepal
**Sharma, 2021a**[303]	125	0	88	NR	NR	01/2021	NA	prospective cohort	other	India

Study	Cases	Index cases	No symptoms	Presymptomatic	Asymptomatic	Publication date	Age class	Study design	Study setting	Country
**Sharma, 2021b**[304]	2	0	2	0	2	02/2021	children	population screening	health care facility	India
**Shen, 2020a**[305]	9	0	2	NR	NR	4/20	children	retrospective cohort	health care facility	China
**Shen, 2020b**[306]	3	1	2	0	2	12/20	mixed	population screening	household	China
**Shi, 2020a**[307]	146	0	66	45	21	8/20	elderly	population screening	nursing facility	USA
**Shi, 2020b**[308]	245	96	1	NR	NR	11/20	mixed	retrospective cohort	health care facility	China
**Shmakov, 2020**[309]	66	0	15	NR	NR	11/20	adults	population screening	other	Russia
**Singer, 2020**[310]	18	0	6	NR	NR	8/20	mixed	retrospective cohort	health care facility	USA
**Singh, 2021**[311]	132	0	86	NR	NR	02/2021	mixed (children and adults)	cross-sectional	other	India
**Son, 2020**[312]	108	0	12	8	4	6/20	mixed	population screening	community	South Korea
**Song, 2020a**[313]	16	4	NR	NR	8	4/20	children	retrospective cohort	health care facility	China
**Soriano-Arandes , 2021**[314]	756	0	400	NR	NR	03/2021	children	prospective cohort	community	Spain
**Soysal, 2020**[315]	237	29	70	0	70	12/20	children	retrospective cohort	health care facility	Turkey
**Stadler, 2021**[316]	26	0	6	NR	NR	02/2021	NA	cross-sectional	health care facility	Switzerland
**Stessel, 2021**[317]	14	0	6	3	3	02/2021	mixed	population screening	health care facility	Belgium

Study	Cases	Index cases	No symptoms	Presymptomatic	Asymptomatic	Publication date	Age class	Study design	Study setting	Country
**Stock, 2020**[318]	19	0	9	2	7	8/20	adults	population screening	health care facility	USA
**Sugano, 2020**[319]	108	1	23	NR	NR	8/20	mixed	retrospective cohort	other	Japan
**Suh, 2020**[320]	161	102	27	17	10	8/20	mixed	retrospective cohort	health care facility	South Korea
**Sun, 2020a**[321]	74	0	22	NR	NR	6/20	children	retrospective cohort	health care facility	China
**Sutton, 2020**[322]	33	0	29	NR	NR	5/20	adults	prospective cohort	other	USA
**Szegedi, 2020**[323]	1	0	1	0	1	12/20	NR	population screening	health care facility	Austria
**Tabata, 2020**[324]	104	0	43	10	33	6/20	mixed	population screening	other	Japan
**Tambe, 2020**[325]	197	140	45	NR	NR	6/20	mixed	population screening	health care facility	India
**Tan-Loh, 2021**[326]	46	0	NR	NR	6	1/21	NA	retrospective cohort	health care facility	Malaysia
**Tan, 2020a**[327]	13	0	2	NR	NR	4/20	children	retrospective cohort	health care facility	China
**Tan, 2020b**[328]	10	0	NR	NR	2	4/20	children	retrospective cohort	health care facility	China
**Tan, 2021**[329]	199	10	146	43	103	03/2021	mixed	retrospective cohort	health care facility	Malaysia
**Tang, 2020b**[330]	752	0	424	NR	NR	10/20	elderly	retrospective cohort	nursing facility	USA
**Tariq, 2020**[331]	67	0	17	NR	NR	8/20	mixed	prospective cohort	health care facility	Saudi Arabia

Study	Cases	Index cases	No symptoms	Presymptomatic	Asymptomatic	Publication date	Age class	Study design	Study setting	Country
**Tawar, 2021**[332]	27	2	19	NR	NR	01/2021	NA	contact tracing	community	India
**Taylor, 2020**[333]	255	38	126	110	16	9/20	mixed	prospective cohort	nursing facility	USA
**Temel, 2020**[334]	81	1	21	5	16	12/20	children	retrospective cohort	health care facility	Turkey
**Thangaraj, 2020**[335]	17	1	16	8	8	6/20	mixed	population screening	community	India
**Thiel, 2020**[336]	43	0	NR	NR	11	10/20	mixed	prospective cohort	community	Liechtenstein
**Thompson, 2020**[337]	51	1	14	NR	NR	10/20	NR	prospective cohort	health care facility	USA
**Tian, 2020a**[338]	262	86	13	NR	NR	2/20	mixed	retrospective cohort	health care facility	China
**Tompkins, 2021**[339]	431	0	350	NR	NR	04/2021	mixed	cross-sectional	other	USA
**Tong, 2020**[340]	6	1	3	0	3	5/20	mixed	population screening	household	China
**Trahan, 2020**[341]	10	0	8	NR	NR	11/20	adults	retrospective cohort	other	Canada
**Tsou, 2020**[342]	100	78	NR	NR	10	7/20	mixed	prospective cohort	community	Taiwan
**Venkataram, 2020**[343]	8	0	7	NR	NR	9/20	mixed	population screening	health care facility	India
**Villa, 2021**[344]	70	0	34	NR	NR	03/2021	NA	cross-sectional	health care facility	USA
**Wadhwa, 2020**[345]	19	0	12	4	8	12/20	adults	population screening	other	USA

**Study**	Cases	Index cases	No symptoms	Presymptomatic	Asymptomatic	Publication date	Age class	Study design	Study setting	Country
**Wang, 2020c**[346]	279	0	NR	NR	63	5/20	mixed	retrospective cohort	community	China
**Wang, 2020e**[347]	12	0	2	0	2	11/20	adults	prospective cohort	health care facility	China
**Wang, 2020f**[348]	1,369	1,112	209	NR	NR	12/20	children	retrospective cohort	health care facility	China
**Wang, 2021**[349]	5	1	2	NR	NR	1/21	mixed	prospective cohort	household	China
**Wanwan, 2020**[350]	189	0	54	NR	NR	6/20	mixed	prospective cohort	household	China
**Wi, 2020**[351]	111	0	25	18	7	7/20	mixed	prospective cohort	community	China
**Wong, 2020a**[352]	138	0	58	42	16	5/20	NR	population screening	community	Brunei
**Wong, 2020b**[353]	5	1	3	1	2	5/20	mixed	prospective cohort	community	Malaysia
**Wu, 2020c**[354]	83	35	NR	NR	8	5/20	mixed	population screening	household	China
**Wu, 2020e**[355]	23	6	3	NR	NR	4/20	children	retrospective cohort	health care facility	China
**Wu, 2020f**[356]	58	19	13	6	7	10/20	NR	retrospective cohort	health care facility	China
**Xi, 2020**[357]	18	14	7	7	0	5/20	mixed	retrospective cohort	health care facility	China
**Xie, 2021**[358]	73	20	4	NR	NR	02/2021	NA	contact tracing	household	China
**Xiong, 2020**[359]	131	0	NR	NR	28	7/20	elderly	population screening	health care facility	China

Study	Cases	Index cases	No symptoms	Presymptomatic	Asymptomatic	Publication date	Age class	Study design	Study setting	Country
**Xu, 2020b**[360]	32	3	11	NR	NR	5/20	children	retrospective cohort	health care facility	China
**Xu, 2020c**[361]	5	0	4	NR	NR	7/20	mixed	prospective cohort	health care facility	China
**Yan, 2020**[362]	65	0	6	NR	NR	4/20	adults	retrospective cohort	other	China
**Yang, 2020a**[363]	6	1	2	0	2	5/20	mixed	prospective cohort	household	China
**Yang, 2020b**[364]	78	0	NR	NR	33	5/20	mixed	prospective cohort	health care facility	China
**Yang, 2020c**[365]	37	0	6	NR	NR	7/20	mixed	prospective cohort	community	China
**Yang, 2020d**[366]	10	0	2	0	2	7/20	adults	population screening	community	China
**Yao-Ling, 2020**[367]	115	10	61	NR	NR	4/20	children	retrospective cohort	health care facility	China
**Yassa, 2020**[368]	23	0	12	NR	NR	7/20	adults	prospective cohort	other	Turkey
**Yau, 2020**[369]	11	1	6	3	3	7/20	elderly	population screening	health care facility	Canada
**Yayla, 2020a**[370]	220	3	55	NR	NR	8/20	children	retrospective cohort	health care facility	Turkey
**Yayla, 2020b**[371]	77	1	19	NR	NR	10/2020	children	retrospective cohort	health care facility	Turkey
**Ye, 2020a**[372]	5	1	4	3	1	3/20	adults	population screening	household	China
**Ye, 2020b**[373]	33	0	4	NR	NR	5/20	mixed	prospective cohort	community	China

Study	Cases	Index cases	No symptoms	Presymptomatic	Asymptomatic	Publication date	Age class	Study design	Study setting	Country
**Yilmaz, 2020**[374]	105	14	59	NR	NR	9/20	children	retrospective cohort	health care facility	Turkey
**Yombi, 2020**[375]	10	0	6	NR	NR	9/20	NR	population screening	other	Belgium
**Yue, 2020**[376]	86	50	16	NR	NR	7/20	mixed	retrospective cohort	health care facility	China
**Zamzuri, 2020**[377]	214	52	84	NR	NR	11/20	mixed	retrospective cohort	community	Malaysia
**Zhan, 2020**[378]	405	343	12	NR	NR	8/20	mixed	retrospective cohort	health care facility	China
**Zhang, 2020a**[379]	5	1	2	1	1	3/20	adults	population screening	household	China
**Zhang, 2020d**[380]	12	0	4	0	4	5/20	mixed	population screening	community	China
**Zhang, 2020e**[381]	10	0	2	0	2	6/20	mixed	prospective cohort	community	China
**Zhang, 2020f**[382]	33	3	8	NR	NR	6/20	children	retrospective cohort	health care facility	China
**Zhang, 2020g**[383]	13	7	4	0	4	7/20	mixed	population screening	household	China
**Zhang, 2020h**[384]	424	0	148	NR	NR	9/20	mixed	retrospective cohort	health care facility	China
**Zhang, 2020i**[385]	22	3	14	0	14	11/20	mixed	prospective cohort	household	China
**Zhao, 2020b**[386]	160	122	38	11	27	8/20	mixed	population screening	health care facility	China
**Zheng, 2020b**[387]	307	132	17	9	8	6/20	mixed	prospective cohort	community	China

**Study**	Cases	Index cases	No symptoms	Presymptomatic	Asymptomatic	Publication date	Age class	Study design	Study setting	Country
**Zhiqiang, 2020**[388]	27	1	1	NR	NR	9/20	NR	prospective cohort	community	China
**Zhou, 2020b**[389]	9	3	5	NR	NR	3/20	children	retrospective cohort	health care facility	China
**Zou, 2020**[390]	18	14	1	NR	NR	2/20	mixed	prospective cohort	community	China

Table S3. Risk of bias assessment with modified ROBINS-I checklist

Study	Item 1*	Item 2*	Item 3*	Item 4*	Item 5*	Item 6*	Item 7*	Overall score
**Abraha, 2021**	low	low	low	low	low	low	low	low
**Al-Qahtani, 2020**	low	low	low	low	low	low	low	low
**Al-Shamsi, 2020b**	low	low	low	low	low	low	low	low
**Almazeedi, 2020**	low	severe	low	low	low	low	low	severe
**Alsharrah, 2020**	low	low	low	low	low	low	low	low
**Alvarado, 2020**	low	low	low	low	low	low	low	low
**An, 2020**	moderate	severe	severe	low	low	low	low	severe
**Anand, 2020b**	low	low	low	low	low	low	severe	severe
**Arima, 2020**	moderate	low	low	low	low	low	low	moderate
**Arons, 2020**	low	low	low	low	low	low	moderate	moderate
**Ashahrani, 2020**	low	low	low	severe	low	low	severe	severe
**Aslam, 2020**	moderate	low	low	low	low	low	low	moderate
**Atalla, 2020**	low	low	low	low	low	low	low	low
**Bae, 2020**	low	severe	low	low	low	low	low	severe
**Beiting, 2021**	low	low	low	moderate	low	low	low	moderate
**Bender, 2020**	low	severe	low	low	low	low	low	severe
**Blain, 2020a**	low	low	low	low	low	low	low	low
**Blain, 2020b**	low	low	low	moderate	low	low	low	moderate
**Blain, 2021**	low	low	low	low	low	low	low	low
**Chan, 2020**	severe	low	low	low	low	low	low	severe
**Chan, 2021**	low	low	low	low	low	low	low	low

Study	Item 1*	Item 2*	Item 3*	Item 4*	Item 5*	Item 6*	Item 7*	Overall score
**Chang, 2020**	low	low	low	severe	low	low	low	severe
**Chau, 2020**	low	low	low	low	low	low	low	low
**Chehrassan, 2020**	low	low	severe	low	moderate	severe	low	severe
**Choudhury, 2020**	severe	low	low	low	low	low	low	severe
**Coppeta, 2020**	low	low	low	low	low	low	low	low
**Corcorann, 2020**	low	low	low	severe	low	low	low	severe
**Cosma, 2020**	low	low	low	severe	low	low	low	severe
**Cruz-Lemini, 2021**	low	severe	low	low	low	low	low	severe
**Danis, 2020**	low	low	low	severe	low	low	severe	severe
**Dora, 2020**	low	low	low	severe	low	low	low	severe
**Du, 2020a**	low	low	low	low	low	low	low	low
**Du, 2020c**	low	low	low	low	low	low	low	low
**Eythorsson, 2020**	low	low	low	severe	low	low	low	severe
**Fusco, 2020**	low	severe	low	moderate	low	low	low	severe
**Gao, 2020**	severe	low	low	moderate	low	low	low	severe
**Golden-Feld, 2020**	low	low	low	low	low	low	low	low
**Gonfiotti, 2020**	low	low	severe	low	low	low	low	severe
**Green, 2021**	low	low	low	low	low	low	low	low
**Grijalva, 2020**	low	low	low	severe	low	low	low	severe
**Gupta, 2020b**	low	low	severe	low	low	low	low	severe
**Gupta, 2021**	low	low	low	low	low	low	low	low
**Gutman, 2021**	low	severe	low	low	low	low	low	severe
**Han, 2020**	low	low	low	low	low	low	low	low

Study	Item 1*	Item 2*	Item 3*	Item 4*	Item 5*	Item 6*	Item 7*	Overall score
**Handal, 2021**	low	low	low	moderate	severe	severe	severe	severe
**Harada, 2020**	low	low	low	low	low	low	low	low
**Hasanoglu, 2020**	low	low	low	low	low	low	low	low
**Hcini, 2020**	low	low	low	severe	low	low	low	severe
**He, 2020a**	low	low	low	low	low	low	low	low
**Hoehl, 2020**	low	low	low	low	low	low	low	low
**Hogan, 2020**	low	severe	low	severe	low	low	low	severe
**Hu, 2020a**	low	severe	low	low	low	low	severe	severe
**Hua, 2020**	low	low	low	low	low	low	low	low
**Huang, 2020a**	low	low	low	low	low	low	low	low
**Huang, 2020b**	low	low	low	low	low	low	low	low
**Huang, 2020c**	low	severe	low	low	low	severe	moderate	severe
**Hung, 2020**	low	low	low	low	low	low	low	low
**Ibrahim, 2020b**	low	low	low	low	low	low	low	low
**Jeong, 2020**	low	low	low	low	low	low	low	low
**Jiang, 2020a**	low	low	low	severe	low	low	low	severe
**Jiang, 2020b**	low	low	low	low	low	low	low	low
**Jiang, 2020d**	low	low	low	low	low	low	low	low
**Jones, 2020**	low	low	low	low	low	low	low	low
**Joshi, 2020**	low	low	low	low	low	low	low	low
**Jung, 2020**	low	low	low	low	low	low	low	low
**Kang, 2020**	low	low	low	low	low	low	low	low
**Karout, 2020**	low	low	low	low	severe	severe	low	severe

Study	Item 1*	Item 2*	Item 3*	Item 4*	Item 5*	Item 6*	Item 7*	Overall score
Kenelly, 2020	low	low	low	low	low	low	low	low
Kimball, 2020	low	low	low	low	low	low	low	low
Kirshblum, 2020	low	low	low	low	low	low	low	low
Kittang, 2020	low	low	low	low	low	low	low	low
Krone, 2021	low	low	low	low	low	low	low	low
Kruger, 2021	low	low	low	low	severe	severe	moderate	severe
Kutsuna, 2020	low	low	low	low	low	low	low	low
Ladhani, 2020	low	low	low	low	low	low	low	low
Lamichhane, 2021	low	low	moderate	severe	low	low	low	severe
Lee, 2020b	low	low	low	low	low	low	low	low
Li, 2020a	low	low	low	low	low	low	low	low
Li, 2020d	low	low	low	low	low	low	low	low
Liao, 2020a	low	low	low	severe	low	low	low	severe
Liu, 2020f	low	low	low	low	low	low	low	low
Liu, 2021	low	low	low	low	low	low	low	low
Lombardi, 2020	low	low	low	low	low	low	low	low
Luo, 2020a	low	low	low	severe	low	low	severe	severe
Luo, 2020b	low	low	low	low	low	low	low	low
Lytras, 2020	low	low	low	severe	low	low	low	severe
Ma, 2020b	low	low	low	low	low	low	low	low
Macartney, 2020	low	severe	low	severe	low	low	low	severe
Malagon-Rojas, 2020	low	low	low	moderate	low	low	low	moderate
Marks, 2021	low	severe	low	severe	low	low	low	severe

Study	Item 1*	Item 2*	Item 3*	Item 4*	Item 5*	Item 6*	Item 7*	Overall score
**Martinez-Fierro, 2020**	severe	low	low	severe	low	low	severe	severe
**Merza, 2020**	low	low	low	low	low	low	low	low
**Meyers, 2020**	low	severe	low	severe	low	low	severe	severe
**Meyers, 2021**	low	severe	low	severe	low	low	low	severe
**Migisha, 2020**	low	low	low	low	low	low	low	low
**Ng, 2020a**	low	low	low	low	low	low	low	low
**Nicolas, 2020**	low	low	low	moderate	low	low	low	moderate
**Njuguna, 2020**	low	low	low	severe	low	low	low	severe
**Olmos, 2021**	low	low	low	low	low	low	low	low
**Park, 2020a**	low	low	low	low	low	low	low	low
**Park, 2020b**	low	low	low	severe	low	low	severe	severe
**Passarelli, 2020a**	low	low	low	low	severe	severe	severe	severe
**Passarelli, 2020b**	severe	low	low	low	low	low	low	severe
**Patel, 2020a**	low	low	low	low	low	low	low	low
**Patil, 2021**	moderate	low	low	low	low	low	low	moderate
**Pavli, 2020a**	low	low	low	severe	severe	low	severe	severe
**Pham, 2020**	low	low	low	severe	severe	severe	moderate	severe
**Plucinski, 2020**	low	low	low	severe	low	low	low	severe
**Pongpirul, 2020a**	severe	low	low	low	low	low	low	severe
**Puylaert, 2020**	low	severe	low	severe	low	low	low	severe
**Qian, 2020**	moderate	low	low	low	low	low	low	moderate
**Qin, 2020**	low	low	low	low	severe	severe	moderate	severe
**Qiu, 2020b**	low	low	low	low	low	low	low	low

Study	Item 1*	Item 2*	Item 3*	Item 4*	Item 5*	Item 6*	Item 7*	Overall score
Rajme-Lopez, 2021	low	low	low	severe	low	low	low	severe
Ralli, 2020	low	low	low	low	low	low	severe	severe
Reale, 2020	low	low	low	low	severe	low	severe	severe
Reid, 2020	low	severe	low	low	severe	severe	severe	severe
Ren, 2021	low	low	low	low	low	low	low	low
Rincon, 2020	low	low	low	low	low	low	low	low
Rivera, 2020	low	severe	low	low	low	low	low	severe
Rivett, 2020	low	low	low	severe	severe	severe	moderate	severe
Roberts, 2021	low	low	low	severe	low	low	low	severe
Saeed, 2020	low	low	low	severe	severe	severe	moderate	severe
Saidy, 2020	low	low	low	low	low	low	low	low
Schwierzeck, 2020	low	low	low	low	low	low	low	low
See, 2020	low	low	low	low	low	low	low	low
Senanayake, 2020	low	low	low	low	low	low	low	low
Setiawaty, 2020	severe	low	low	low	low	low	low	severe
Sharma, 2021b	low	low	low	low	moderate	moderate	moderate	moderate
Shen, 2020b	low	low	low	severe	low	low	low	severe
Shi, 2020	low	low	severe	low	low	low	severe	severe
Son, 2020	low	low	low	moderate	severe	severe	moderate	severe
Song, 2020a	low	low	severe	low	low	low	low	severe
Soysal, 2020	low	low	low	low	low	low	low	low
Stessel, 2021	low	low	low	severe	low	low	low	severe
Stock, 2020	low	severe	low	low	low	low	low	severe

Study	Item 1*	Item 2*	Item 3*	Item 4*	Item 5*	Item 6*	Item 7*	Overall score
Suh, 2020	low	low	low	low	low	low	low	low
Szegedi, 2020	low	low	low	low	low	low	low	low
Tabata, 2020	low	low	low	low	severe	severe	low	severe
Tan-Loh, 2021	low	low	low	low	low	low	low	low
Tan, 2020b	low	low	low	low	low	low	low	low
Tan, 2021	low	low	low	low	low	low	low	low
Taylor, 2020	low	low	low	low	low	low	severe	severe
Temel, 2020	low	low	low	severe	low	severe	low	severe
Thangaraj, 2020	low	low	low	low	low	low	low	low
Thiel, 2020	low	low	low	severe	low	low	low	severe
Tong, 2020	low	low	low	severe	low	low	low	severe
Tsou, 2020	low	low	low	low	low	low	low	low
Wadhwa, 2020	low	low	low	severe	low	low	low	severe
Wang, 2020c	low	severe	low	low	severe	severe	severe	severe
Wang, 2020e	low	low	severe	low	low	low	low	severe
Wi, 2020	low	low	low	low	low	low	low	low
Wong, 2020a	low	low	low	low	low	low	severe	severe
Wong, 2020b	severe	low	low	low	low	low	low	severe
Wu, 2020c	low	low	low	low	low	low	low	low
Wu, 2020f	low	low	low	low	severe	severe	low	severe
Xi, 2020	low	low	low	low	low	low	low	low
Xiong, 2020	low	low	low	low	low	low	severe	severe
Yang, 2020a	severe	low	low	low	low	low	low	severe

Study	Item 1*	Item 2*	Item 3*	Item 4*	Item 5*	Item 6*	Item 7*	Overall score
**Yang, 2020b**	low	low	low	low	severe	severe	severe	severe
**Yang, 2020d**	severe	low	low	low	low	low	severe	severe
**Yau, 2020**	severe	low	low	low	low	low	low	severe
**Ye, 2020a**	low	low	low	low	severe	severe	low	severe
**Zhang, 2020a**	low	low	low	low	severe	severe	low	severe
**Zhang, 2020d**	low	severe	low	low	low	low	low	severe
**Zhang, 2020e**	severe	low	low	severe	low	low	low	severe
**Zhang, 2020g**	low	low	low	low	low	low	low	low
**Zhang, 2020i**	severe	low	low	low	low	low	low	severe
**Zhao, 2020b**	low	low	low	low	low	low	low	low
**Zheng, 2020b**	low	low	low	low	low	low	low	low

*(1) Enrollment of all patients satisfying the criteria for inclusion, (2) enrollment of cases regardless of symptom status, (3) confirmation of cases using RT-PCR, (4) symptoms monitored by clinicians rather than self-reporting, (5) symptom assessment at the end of the follow-up period, (6) symptom follow-up duration of at least seven days, (7) loss to follow-up less than 5%.

SI References

1 Abeysuriya S, Wasif S, Counihan C, *et al.* Universal screening for SARS-CoV-2 in pregnant women at term admitted to an East London maternity unit. *Eur J Obstet Gynecol Reprod Biol* 2020; **252**: 444–6.

2 Abraha HE, Gessesse Z, Gebrecherkos T, *et al.* Clinical features and risk factors associated with morbidity and mortality among patients with COVID-19 in northern Ethiopia. *Int J Infect Dis* 2021; **105**: 776–83.

3 Adetola HH, Ishola D, Afolabi MO, *et al.* Clinical presentations and management of COVID-19 infected children seen in a district health facility in Kambia, northern Sierra Leone. *Pan Afr Med J* 2020; **37**: 28.

4 Adorni F, Prinelli F, Bianchi F, *et al.* Self-reported symptoms of SARS-CoV-2 infection in a non-hospitalized population: results from the large Italian web-based EPICOVID19 cross-sectional survey. *JMIR Public Health Surveill* 2020; published online July 10. DOI:10.2196/21866.

5 Aherfi S, Gautret P, Chaudet H, Raoult D, La Scola B. Clusters of COVID-19 associated with Purim celebration in the Jewish community in Marseille, France, March 2020. *Int J Infect Dis* 2020; **100**: 88–94.

6 Al-Qahtani M, AlAli S, AbdulRahman A, Salman Alsayyad A, Otoom S, Atkin SL. The prevalence of asymptomatic and symptomatic COVID-19 in a cohort of quarantined subjects. *Int J Infect Dis* 2020; **102**: 285–8.

7 Alshami A, Alattas R, Anan H, Alhalimi A, Alfaraj A, Al Qahtani H. Silent disease and loss of taste and smell are common manifestations of SARS-COV-2 infection in a quarantine facility: Saudi Arabia. *PLoS One* 2020; **15**: e0241258.

8 Albalate M, Arribas P, Torres E, *et al.* Alta prevalencia de COVID-19 asintomático en hemodiálisis. Aprendiendo día a día el primer mes de pandemia de COVID-19. *Nefrología* 2020; **40**: 279–86.

9 Almadhi MA, Abdulrahman A, Sharaf SA, *et al.* The high prevalence of asymptomatic SARS-CoV-2 infection reveals the silent spread of COVID-19. *Int J Infect Dis* 2021; **105**: 656–61.

10 Almazeedi S, Al-Youha S, Jamal MH, *et al.* Characteristics, risk factors and outcomes among the first consecutive 1096 patients diagnosed with COVID-19 in Kuwait. *EClinicalMedicine* 2020; **24**: 100448.

11 Alsharrah D, Alhaddad F, Alyaseen M, *et al.* Clinical characteristics of pediatric SARS-CoV-2 infection and coronavirus disease 2019 (COVID-19) in Kuwait. *J Med Virol* 2020; published online Nov 20. DOI:10.1002/jmv.26684.

12 Alvarado GR, Pierson BC, Teemer ES, Gama HJ, Cole RD, Jang SS. Symptom characterization and outcomes of sailors in isolation after a COVID-19 outbreak on a US

aircraft carrier. *JAMA Netw Open* 2020; **3**: e2020981.

13 An P, Song P, Wang Y, Liu B. Asymptomatic Patients with Novel Coronavirus Disease (COVID-19). *Balkan Med J* 2020; published online April 13. DOI:10.4274/balkanmedj.galenos.2020.2020.4.20.

14 Anand P, Yadav A, Debata P, Bachani S, Gupta N, Gera R. Clinical profile, viral load, management and outcome of neonates born to COVID 19 positive mothers: a tertiary care centre experience from India. *Eur J Pediatr* 2020; published online Sept 10. DOI:10.1007/s00431-020-03800-7.

15 Andrikopoulou M, Madden N, Wen T, *et al.* Symptoms and Critical Illness Among Obstetric Patients With Coronavirus Disease 2019 (COVID-19) Infection. *Obstet Gynecol* 2020; **136**: 291–9.

16 Arima Y, Shimada T, Suzuki M, *et al.* Severe Acute Respiratory Syndrome Coronavirus 2 Infection among Returnees to Japan from Wuhan, China, 2020. *Emerg Infect Dis* 2020; **26**. DOI:10.3201/eid2607.200994.

17 Arnold FW, Bishop S, Oppy L, Scott L, Stevenson G. Surveillance testing reveals a significant proportion of hospitalized patients with SARS-CoV-2 are asymptomatic. *Am J Infect Control* 2021; published online Jan 9. DOI:10.1016/j.ajic.2021.01.005.

18 Arons MM, Hatfield KM, Reddy SC, *et al.* Presymptomatic SARS-CoV-2 Infections and Transmission in a Skilled Nursing Facility. *N Engl J Med* 2020; published online April 24. DOI:10.1056/NEJMoa2008457.

19 Alshahrani MS, Alnimr A, Alnassri S, Alfarag S, Aljehani Y, Alabdali M. Prevalence of the SARS-CoV-2 infection among post-quarantine healthcare workers. *J Multidiscip Healthc* 2020; **13**: 1927–36.

20 Aslam A, Singh J, Robilotti E, *et al.* SARS CoV-2 surveillance and exposure in the perioperative setting with universal testing and personal protective equipment (PPE) policies. *Clin Infect Dis* 2020; published online Oct 22. DOI:10.1093/cid/ciaa1607.

21 Atakla HG, Noudohounsi MMUD, Salami AY, *et al.* COVID-19 infection in pediatric subjects: study of 36 cases in Conakry. *Pan Afr Med J* 2020; **37**: 42.

22 Atalla E, Zhang R, Shehadeh F, *et al.* Clinical presentation, course, and risk factors associated with mortality in a severe outbreak of COVID-19 in Rhode Island, USA, April-June 2020. *Pathogens* 2020; **10**: 8.

23 Bae SH, Shin H, Koo H-Y, Lee SW, Yang JM, Yon DK. Asymptomatic Transmission of SARS-CoV-2 on Evacuation Flight. *Emerg Infect Dis* 2020; **26**. DOI:10.3201/eid2611.203353.

24 Baggett TP, Keyes H, Sporn N, Gaeta JM. Prevalence of SARS-CoV-2 Infection in Residents of a Large Homeless Shelter in Boston. *JAMA* 2020; published online April 27.

DOI:10.1001/jama.2020.6887.

25 Bai Y, Yao L, Wei T, *et al.* Presumed Asymptomatic Carrier Transmission of COVID-19. *JAMA* 2020; published online Feb 21. DOI:10.1001/jama.2020.2565.

26 Shaoli B, Jianyun W, Yingquan Z, *et al.* Analysis of the first cluster of cases in a family of novel coronavirus pneumonia in Gansu Province. *Chinese Journal of Preventive Medicine* 2020; **54**: E005–E005.

27 Bayle C, Cantin D, Vidal J-S, *et al.* Asymptomatic SARS COV-2 carriers among nursing home staff: A source of contamination for residents? *Infect Dis Now* 2021; published online Jan 18. DOI:10.1016/j.idnow.2020.11.008.

28 Beiting KJ, Huisingh-Scheetz M, Walker J, *et al.* Management and outcomes of a COVID-19 outbreak in a nursing home with predominantly Black residents. *J Am Geriatr Soc* 2021; published online March 19. DOI:10.1111/jgs.17126.

29 Bender WR, Hirshberg A, Coutifaris P, Acker AL, Srinivas SK. Universal Testing for SARS-CoV-2 in Two Philadelphia Hospitals: Carrier Prevalence and Symptom Development Over Two Weeks. *Am J Obstet Gynecol MFM* 2020; : 100226.

30 Bender JK, Brandl M, Höhle M, Buchholz U, Zeitlmann N. Analysis of Asymptomatic and Presymptomatic Transmission in SARS-CoV-2 Outbreak, Germany, 2020. *Emerg Infect Dis* 2021; **27**. DOI:10.3201/eid2704.204576.

31 Berghoff AS, Gansterer M, Bathke AC, *et al.* SARS-CoV-2 Testing in Patients With Cancer Treated at a Tertiary Care Hospital During the COVID-19 Pandemic. *J Clin Oncol* 2020; : JCO2001442.

32 Berry M, Wang A, Clark SM, *et al.* Clinical Stratification of Pregnant COVID-19 Patients based on Severity: A Single Academic Center Experience. *Am J Perinatol* 2021; **38**: 515–22.

33 Bi Q, Wu Y, Mei S, *et al.* Epidemiology and transmission of COVID-19 in 391 cases and 1286 of their close contacts in Shenzhen, China: a retrospective cohort study. *Lancet Infect Dis* 2020; **20**: 911–9.

34 Bigelow BF, Tang O, Barshick B, *et al.* Outcomes of Universal COVID-19 Testing Following Detection of Incident Cases in 11 Long-term Care Facilities. *JAMA Intern Med* 2021; **181**: 127–9.

35 Blain H, Rolland Y, Tuaillon E, *et al.* Efficacy of a Test-Retest Strategy in Residents and Health Care Personnel of a Nursing Home Facing a COVID-19 Outbreak. *J Am Med Dir Assoc* 2020; **21**: 933–6.

36 Blain H, Rolland Y, Benetos A, *et al.* Atypical clinical presentation of COVID-19 infection in residents of a long-term care facility. *Eur Geriatr Med* 2020; published online Oct 6. DOI:10.1007/s41999-020-00352-9.

37 Blain H, Gamon L, Tuaillon E, *et al.* Atypical symptoms, SARS-CoV-2 test results and immunisation rates in 456 residents from eight nursing homes facing a COVID-19 outbreak. *Age Ageing* 2021; **50**: 641–8.

38 Blitz MJ, Rochelson B, Rausch AC, *et al.* Universal testing for coronavirus disease 2019 in pregnant women admitted for delivery: prevalence of peripartum infection and rate of asymptomatic carriers at four New York hospitals within an integrated healthcare system. *American Journal of Obstetrics & Gynecology MFM* 2020; : 100169.

39 Blumberg TJ, Adler AC, Lin EE, *et al.* Universal Screening for COVID-19 in Children Undergoing Orthopaedic Surgery: A Multicenter Report. *J Pediatr Orthop* 2020; **40**: e990–3.

40 Brandstetter S, Roth S, Harner S, *et al.* Symptoms and immunoglobulin development in hospital staff exposed to a SARS-CoV-2 outbreak. *Pediatr Allergy Immunol* 2020; published online May 15. DOI:10.1111/pai.13278.

41 Cabraal MNS, Samarawickrama RIU, Kodithuwakku KARR, Viswakula SD, Lantra SR. Nationwide descriptive study of COVID-19 in children below the age of 14 years in Sri Lanka. *Sri Lanka J Child Health* 2021; **50**: 103.

42 Camara E, Barry IK, Diallo FB, *et al.* Profil épidémiologique et clinique des enfants atteints de la maladie à Corona Virus (COVID-19) au Centre de Traitement des Epidémies et Prévention des Infections (CTEPI) du CHU de Donka à Conakry. *Pan Afr Med J* 2020; **37**. DOI:10.11604/pamj.2020.37.363.26211.

43 Campbell KH, Tornatore JM, Lawrence KE, *et al.* Prevalence of SARS-CoV-2 Among Patients Admitted for Childbirth in Southern Connecticut. *JAMA* 2020; published online May 26. DOI:10.1001/jama.2020.8904.

44 Cardona-Hernandez R, Cherubini V, Iafusco D, Schiaffini R, Luo X, Maahs DM. Children and youth with diabetes are not at increased risk for hospitalization due to COVID-19. *Pediatr Diabetes* 2020; published online Nov 17. DOI:10.1111/pedi.13158.

45 Carroll C, Conway R, O'Donnell D, *et al.* Routine testing of close contacts of confirmed COVID-19 cases – National COVID-19 Contact Management Programme, Ireland, May to August 2020. *Public Health* 2020; published online Oct. DOI:10.1016/j.puhe.2020.10.008.

46 Cattelan AM, Sasset L, Di Meco E, *et al.* An Integrated Strategy for the Prevention of SARS-CoV-2 Infection in Healthcare Workers: A Prospective Observational Study. *Int J Environ Res Public Health* 2020; **17**. DOI:10.3390/ijerph17165785.

47 Cesilia C, Sudarmaji S, Setiabudi D, Nataprawira HM. Case report of a COVID-19 family cluster originating from a boarding school. *Paediatr Indones* 2021; **61**: 53–60.

48 Chan JF-W, Yuan S, Kok K-H, *et al.* A familial cluster of pneumonia associated with the 2019 novel coronavirus indicating person-to-person transmission: a study of a family cluster. *Lancet* 2020; **0**. DOI:10.1016/S0140-6736(20)30154-9.

49 Chan J, Burke K, Bedard R, *et al.* COVID-19 in the New York City Jail System: Epidemiology and Health Care Response, March-April 2020. *Public Health Rep* 2021; **136**: 375–83.

50 Chang MC, Seo W-S, Park D, Hur J. Analysis of SARS-CoV-2 Screening Clinic (Including Drive-Through System) Data at a Single University Hospital in South Korea from 27 January 2020 to 31 March 2020 during the COVID-19 Outbreak. *Healthcare (Basel)* 2020; **8**. DOI:10.3390/healthcare8020145.

51 Chau NVV, Thanh Lam V, Thanh Dung N, *et al.* The natural history and transmission potential of asymptomatic SARS-CoV-2 infection. *Clin Infect Dis* 2020; published online June 4. DOI:10.1093/cid/ciaa711.

52 Chehrassan M, Ebrahimpour A, Ghandhari H, *et al.* Management of Spine Trauma in COVID-19 Pandemic: A Preliminary Report. *Arch Bone Jt Surg* 2020; **8**: 270–6.

53 Chekhlabi N, Kettani CE, Haoudar A, *et al.* The epidemiological and clinical profile of COVID-19 in children: Moroccan experience of the Cheikh Khalifa University Center. *Pan Afr Med J* 2020; **35**. DOI:10.11604/pamj.supp.2020.35.2.23571.

54 Chen P, Zhang Y, Wen Y, *et al.* Clinical and Demographic Characteristics of Cluster Cases and Sporadic Cases of Coronavirus Disease 2019 (COVID-19) in 141 Patients in the Main District of Chongqing, China, Between January and February 2020. *Med Sci Monit* 2020; **26**: e923985.

55 Chen P, Zhang Y, Wen Y, *et al.* Epidemiological and clinical characteristics of 136 cases of COVID-19 in main district of Chongqing. *J Formos Med Assoc* 2020; **119**: 1180–4.

56 Chen Y, Wang AH, Yi B, *et al.* [Epidemiological characteristics of infection in COVID-19 close contacts in Ningbo city]. *Zhonghua Liu Xing Bing Xue Za Zhi* 2020; **41**: 667–71.

57 Chen J, He H, Cheng W, *et al.* Potential transmission of SARS-CoV-2 on a flight from Singapore to Hangzhou, China: An epidemiological investigation. *Travel Med Infect Dis* 2020; **36**: 101816.

58 Cheng H-Y, Jian S-W, Liu D-P, *et al.* Contact Tracing Assessment of COVID-19 Transmission Dynamics in Taiwan and Risk at Different Exposure Periods Before and After Symptom Onset. *JAMA Intern Med* 2020; published online May 1. DOI:10.1001/jamainternmed.2020.2020.

59 Chiu C-H, Yeh K-M, Su Y-S, Chiu S-K, Lin J-C, Yang Y-S. Familial cluster of pneumonia and asymptomatic cases of COVID-19 in Taiwan. *J Formos Med Assoc* 2020; published online May 21. DOI:10.1016/j.jfma.2020.05.023.

60 Choudhury A, Reddy GS, Venishetty S, *et al.* COVID-19 in Liver Transplant Recipients - A Series with Successful Recovery. *J Clin Transl Hepatol* 2020; **8**: 467–73.

61 Clarke C, Prendecki M, Dhutia A, *et al.* High Prevalence of Asymptomatic COVID-19

Infection in Hemodialysis Patients Detected Using Serologic Screening. *J Am Soc Nephrol* 2020; published online July 30. DOI:10.1681/ASN.2020060827.

62 Coppeta L, Somma G, Ippoliti L, *et al.* Contact screening for healthcare workers exposed to patients with COVID-19. *Int J Environ Res Public Health* 2020; **17**: 9082.

63 Corcorran MA, Olin S, Rani G, *et al.* Prolonged Persistence of PCR-detectable Virus During an Outbreak of SARS-CoV-2 in an Inpatient Geriatric Psychiatry Unit in King County, Washington. *Am J Infect Control* 2020; published online Aug 19. DOI:10.1016/j.ajic.2020.08.025.

64 Cosma S, Borella F, Carosso A, *et al.* The "scar" of a pandemic: cumulative incidence of COVID-19 during the first trimester of pregnancy. *J Med Virol* 2020; published online July 7. DOI:10.1002/jmv.26267.

65 Crameri GAG, Bielecki M, Züst R, Buehrer TW, Stanga Z, Deuel JW. Reduced maximal aerobic capacity after COVID-19 in young adult recruits, Switzerland, May 2020. *Euro Surveill* 2020; **25**. DOI:10.2807/1560-7917.ES.2020.25.36.2001542.

66 Cruz-Lemini M, Ferriols Perez E, de la Cruz Conty ML, *et al.* Obstetric Outcomes of SARS-CoV-2 Infection in Asymptomatic Pregnant Women. *Viruses* 2021; **13**. DOI:10.3390/v13010112.

67 D'Ambrosi F, Rossi G, Soldavini CM, *et al.* Management of gestational diabetes in women with a concurrent Sars-Cov-2 infection, experience of a single center in Northern Italy. *Int J Gynaecol Obstet* 2020; published online Oct 25. DOI:10.1002/ijgo.13434.

68 Stock da Cunha T, Gomá-Garcés E, Avello A, *et al.* The Spectrum of Clinical and Serological Features of COVID-19 in Urban Hemodialysis Patients. *J Clin Med Res* 2020; **9**. DOI:10.3390/jcm9072264.

69 Silva JH da, Oliveira EC de, Hattori TY, Lemos ERS de, Terças-Trettel ACP. Descrição de um cluster da COVID-19: o isolamento e a testagem em assintomáticos como estratégias de prevenção da disseminação local em Mato Grosso, 2020. *Epidemiol Serv Saúde* 2020; **29**. DOI:10.5123/s1679-49742020000400005.

70 Danis K, Epaulard O, Bénet T, *et al.* Cluster of Coronavirus Disease 2019 (COVID-19) in the French Alps, February 2020. *Clin Infect Dis* 2020; **71**: 825–32.

71 Dbeibo L, Kuebler K, Keen A, *et al.* Assessment of a Universal Preprocedural Screening Program for COVID-19. *Infect Control Hosp Epidemiol* 2021; : 1–9.

72 Jesus MCS de, Lima AGA, Santos VS, *et al.* Family COVID-19 cluster analysis of an infant without respiratory symptoms. *Rev Soc Bras Med Trop* 2020; **53**: e20200494.

73 Dhuyvetter A, Cejtin HE, Adam M, Patel A. Coronavirus disease 2019 in pregnancy: The experience at an urban safety net hospital. *J Community Health* 2020; published online Oct 31. DOI:10.1007/s10900-020-00940-7.

74 Díaz-Corvillón P, Mönckeberg M, Barros A, *et al.* Routine screening for SARS CoV-2 in unselected pregnant women at delivery. *PLoS One* 2020; **15**: e0239887.

75 Dixon BE, Wools-Kaloustian KK, Fadel WF, *et al.* Symptoms and symptom clusters associated with SARS-CoV-2 infection in community-based populations: Results from a statewide epidemiological study. *PLoS One* 2021; **16**: e0241875.

76 Donahue M, Sreenivasan N, Stover D, *et al.* Notes from the Field: Characteristics of Meat Processing Facility Workers with Confirmed SARS-CoV-2 Infection - Nebraska, April-May 2020. *MMWR Morb Mortal Wkly Rep* 2020; **69**: 1020–2.

77 Dora AV, Winnett A, Jatt LP, *et al.* Universal and Serial Laboratory Testing for SARS-CoV-2 at a Long-Term Care Skilled Nursing Facility for Veterans - Los Angeles, California, 2020. *MMWR Morb Mortal Wkly Rep* 2020; **69**: 651–5.

78 Du W, Yu J, Wang H, *et al.* Clinical characteristics of COVID-19 in children compared with adults in Shandong Province, China. *Infection* 2020; **48**: 445–52.

79 Du H, Dong X, Zhang J-J, *et al.* Clinical characteristics of 182 pediatric COVID-19 patients with different severities and allergic status. *Allergy* 2020; published online June 10. DOI:10.1111/all.14452.

80 Du W, Yu J, Liu X, Chen H, Lin L, Li Q. Persistence of SARS-CoV-2 virus RNA in feces: A case series of children. *J Infect Public Health* 2020; **13**: 926–31.

81 Duan W, Xiuli J, Feng X, *et al.* Clinical analysis of 31 cases of 2019 novel coronavirus infection in children from six provinces (autonomous region) of northern China. *Chinese Journal of Pediatrics* 2020; **58**: E011–E011.

82 Edlow AG, Li JZ, Collier A-RY, *et al.* Assessment of maternal and neonatal SARS-CoV-2 viral load, transplacental antibody transfer, and placental pathology in pregnancies during the COVID-19 pandemic. *JAMA Netw Open* 2020; **3**: e2030455.

83 El-Sokkary RH, El-Kholy A, Mohy Eldin S, *et al.* Characteristics and predicting factors of Corona Virus Disease-2019 (COVID-19) among healthcare providers in a developing country. *PLoS One* 2021; **16**: e0245672.

84 Eythorsson E, Helgason D, Ingvarsson RF, *et al.* Clinical spectrum of coronavirus disease 2019 in Iceland: population based cohort study. *BMJ* 2020; **371**: m4529.

85 Fakhim H, Nasri E, Aboutalebian S, *et al.* Asymptomatic carriers of coronavirus disease 2019 among healthcare workers in Isfahan, Iran. *Future Virol* 2021; **16**: 93–8.

86 Felice C, Di Tanna GL, Zanus G, Grossi U. Impact of COVID-19 Outbreak on Healthcare Workers in Italy: Results from a National E-Survey. *J Community Health* 2020; **45**: 675–83.

87 Fusco FM, Pisaturo M, Iodice V, *et al.* COVID-19 among healthcare workers in a specialist infectious diseases setting in Naples, Southern Italy: results of a cross-sectional surveillance

study. *J Hosp Infect* 2020; **105**: 596–600.

88 Gao Y, Shi C, Chen Y, *et al.* A cluster of the Corona Virus Disease 2019 caused by incubation period transmission in Wuxi, China. *J Infect* 2020; **80**: 666–70.

89 Gaur A, Meena SK, Bairwa R, *et al.* Clinico-radiological Presentation of COVID-19 Patients at a Tertiary Care Center at Bhilwara Rajasthan, India. *J Assoc Physicians India* 2020; **68**: 29–33.

90 Goldenfeld M, Nir-Paz R, Segal G, *et al.* Characteristics of Clinically Asymptomatic Patients with SARS-CoV-2 Infections, Case Series. *Prehosp Disaster Med* 2021; **36**: 125–8.

91 Goldfarb IT, Diouf K, Barth WH, *et al.* Universal SARS-CoV-2 testing on admission to the labor and delivery unit: Low prevalence among asymptomatic obstetric patients. *Infect Control Hosp Epidemiol* 2020; : 1–2.

92 Gonfiotti A, Gatteschi L, Salvicchi A, Bongiolatti S, Lavorini F, Voltolini L. Clinical courses and outcomes of five patients with primary lung cancer surgically treated while affected by Severe acute respiratory syndrome coronavirus 2. *Eur J Cardiothorac Surg* 2020; **58**: 598–604.

93 Gong X, Xiao W, Cui Y, *et al.* Three infection clusters related with potential pre-symptomatic transmission of coronavirus disease (COVID-19), Shanghai, China, January to February 2020. *Euro Surveill* 2020; **25**. DOI:10.2807/1560-7917.ES.2020.25.33.2000228.

94 Graham NSN, Junghans C, Downes R, *et al.* SARS-CoV-2 infection, clinical features and outcome of COVID-19 in United Kingdom nursing homes. *J Infect* 2020; **81**: 411–9.

95 Grechukhina O, Greenberg V, Lundsberg LS, *et al.* Coronavirus Disease 2019 (COVID-19) pregnancy outcomes in a racially and ethnically diverse population. *Am J Obstet Gynecol MFM* 2020; : 100246.

96 Green R, Tulloch JSP, Tunnah C, *et al.* COVID-19 testing in outbreak free care homes: What are the public health benefits? *J Hosp Infect* 2021; published online Jan 13. DOI:10.1016/j.jhin.2020.12.024.

97 Greiner J, Götz M, Malner-Wagner W, *et al.* Characteristics and mechanisms to control a COVID-19 outbreak on a leukemia and stem cell transplantation unit. *Cancer Med* 2020; published online Dec 12. DOI:10.1002/cam4.3612.

98 Grijalva CG, Rolfes MA, Zhu Y, *et al.* Transmission of SARS-COV-2 infections in households — Tennessee and Wisconsin, April–September 2020. *MMWR Morb Mortal Wkly Rep* 2020; **69**. DOI:10.15585/mmwr.mm6944e1.

99 Gruskay JA, Dvorzhinskiy A, Konnaris MA, *et al.* Universal Testing for COVID-19 in Essential Orthopaedic Surgery Reveals a High Percentage of Asymptomatic Infections. *J Bone Joint Surg Am* 2020; published online June 8. DOI:10.2106/JBJS.20.01053.

100 Gudbjartsson DF, Helgason A, Jonsson H, *et al.* Spread of SARS-CoV-2 in the Icelandic population. *N Engl J Med* 2020; **382**: 2302–15.

101 Guo C-X, He L, Yin J-Y, *et al.* Epidemiological and clinical features of pediatric COVID-19. *BMC Med* 2020; **18**: 250.

102 Gupta N, Saravu K, Varma M, Pm A, Shetty S, Umakanth S. Transmission of SARS-CoV-2 infection by children: A study of contacts of index paediatric cases in India. *J Trop Pediatr* 2020; published online Dec 5. DOI:10.1093/tropej/fmaa081.

103 Gupta N, John A, Kokkottil MS, Varma M, Umakanth S, Saravu K. Clinical profile and outcomes of asymptomatic vs. symptomatic travellers diagnosed with COVID-19: An observational study from a coastal town in South India. *Drug Discov Ther* 2021; **15**: 1–8.

104 Gutman MJ, Patel MS, Vannello C, *et al.* What was the Prevalence of COVID-19 in Asymptomatic Patients Undergoing Orthopaedic Surgery in One Large United States City Mid-pandemic? *Clin Orthop Relat Res* 2021; published online March 12. DOI:10.1097/CORR.0000000000001697.

105 Han MS, Choi EH, Chang SH, *et al.* Clinical Characteristics and Viral RNA Detection in Children With Coronavirus Disease 2019 in the Republic of Korea. *JAMA Pediatr* 2020; published online Aug 28. DOI:10.1001/jamapediatrics.2020.3988.

106 Han T. Outbreak investigation: transmission of COVID-19 started from a spa facility in a local community in Korea. *Epidemiol Health* 2020; **42**: e2020056.

107 Han T, Hua L, He S, *et al.* The epidemiological characteristics of cluster transmission of coronavirus disease 2019 (COVID-19): a multi-center study in Jiangsu Province. *Am J Transl Res* 2020; **12**: 6434–44.

108 Handal N, Whitworth J, Blomfeldt A, *et al.* Comparison of SARS-CoV-2 infections in healthcare workers with high and low exposures to Covid-19 patients in a Norwegian University Hospital. *Infect Dis* 2021; **53**: 420–9.

109 Harada S, Uno S, Ando T, *et al.* Control of a nosocomial outbreak of COVID-19 in a university hospital. *Open Forum Infect Dis* 2020; **7**: ofaa512.

110 Hasanoglu I, Korukluoglu G, Asilturk D, *et al.* Higher viral loads in asymptomatic COVID-19 patients might be the invisible part of the iceberg. *Infection* 2020; published online Nov 24. DOI:10.1007/s15010-020-01548-8.

111 Hcini N, Maamri F, Picone O, *et al.* Maternal, fetal and neonatal outcomes of large series of SARS-CoV-2 positive pregnancies in peripartum period: A single-center prospective comparative study. *Eur J Obstet Gynecol Reprod Biol* 2020; **257**: 11–8.

112 He X, Lau EHY, Wu P, *et al.* Temporal dynamics in viral shedding and transmissibility of COVID-19. *Nat Med* 2020; published online April 15. DOI:10.1038/s41591-020-0869-5.

113 He M, Wang C, Xu L, *et al.* Epidemiological and clinical characteristics of 35 children with COVID-19 in Beijing, China. *Pediatr Investig* 2020; **4**: 230–5.

114 Hempel L, Piehler A, Pfaffl MW, *et al.* SARS-CoV-2 infections in cancer outpatients-Most infected patients are asymptomatic carriers without impact on chemotherapy. *Cancer Med* 2020; published online Oct 6. DOI:10.1002/cam4.3435.

115 Herraiz I, Folgueira D, Villalaín C, Forcén L, Delgado R, Galindo A. Universal screening for SARS-CoV-2 before labor admission during Covid-19 pandemic in Madrid. *J Perinat Med* 2020; published online July 20. DOI:10.1515/jpm-2020-0236.

116 Hijnen D, Marzano AV, Eyerich K, *et al.* SARS-CoV-2 Transmission from Presymptomatic Meeting Attendee, Germany. *Emerg Infect Dis* 2020; **26**: 1935–7.

117 Hoehl S, Rabenau H, Berger A, *et al.* Evidence of SARS-CoV-2 Infection in Returning Travelers from Wuhan, China. *N Engl J Med* 2020; **382**: 1278–80.

118 Hogan CA, Gombar S, Wang H, *et al.* Large-scale testing of asymptomatic healthcare personnel for severe acute respiratory syndrome Coronavirus 2. *Emerg Infect Dis* 2021; **27**: 250–4.

119 Hong J-M, Hu L-H, Zhong Q-S, *et al.* Epidemiological characteristics and clinical features of patients infected with the COVID-19 virus in Nanchang, Jiangxi, China. *Front Med (Lausanne)* 2020; **7**: 571069.

120 Hu Z, Song C, Xu C, *et al.* Clinical characteristics of 24 asymptomatic infections with COVID-19 screened among close contacts in Nanjing, China. *Sci China Life Sci* 2020; **63**: 706–11.

121 Hu X, Gao J, Luo X, *et al.* Severe Acute Respiratory Syndrome Coronavirus 2 (SARS-CoV-2) Vertical Transmission in Neonates Born to Mothers With Coronavirus Disease 2019 (COVID-19) Pneumonia. *Obstet Gynecol* 2020; **136**: 65–7.

122 Hu S, Wang W, Wang Y, *et al.* Infectivity, susceptibility, and risk factors associated with SARS-CoV-2 transmission under intensive contact tracing in Hunan, China. *Nat Commun* 2021; **12**: 1533.

123 Hua C-Z, Miao Z-P, Zheng J-S, *et al.* Epidemiological features and viral shedding in children with SARS-CoV-2 infection. *J Med Virol* 2020; published online June 15. DOI:10.1002/jmv.26180.

124 Huang L, Jiang J, Li X, Zhou Y, Xu M, Zhou J. Initial CT imaging characters of an imported family cluster of COVID-19. *Clin Imaging* 2020; **65**: 78–81.

125 Huang K, Zhang J, Wu W, *et al.* A retrospective analysis of the epidemiology, clinical manifestations, and imaging characteristics of familial cluster-onset COVID-19. *Ann Transl Med* 2020; **8**: 747.

126 Huang Q, Hu S, Ran F-M, *et al.* Asymptomatic COVID-19 infection in patients with cancer at a cancer-specialized hospital in Wuhan, China - Preliminary results. *Eur Rev Med Pharmacol Sci* 2020; **24**: 9760–4.

127 Huete-Pérez JA, Cabezas-Robelo C, Páiz-Medina L, Hernández-Álvarez CA, Quant-Durán C, McKerrow JH. First report on prevalence of SARS-CoV-2 infection among health-care workers in Nicaragua. *PLoS One* 2021; **16**: e0246084.

128 Hung IF-N, Cheng VC-C, Li X, *et al.* SARS-CoV-2 shedding and seroconversion among passengers quarantined after disembarking a cruise ship: a case series. *Lancet Infect Dis* 2020; published online June 12. DOI:10.1016/S1473-3099(20)30364-9.

129 Ibrahim O, Suleiman BM, Sanda A, *et al.* COVID-19 in children: a case series from Nigeria. *Pan Afr Med J* 2020; **35**. DOI:10.11604/pamj.supp.2020.35.2.23597.

130 Ing AJ, Cocks C, Green JP. COVID-19: in the footsteps of Ernest Shackleton. *Thorax* 2020; **75**: 693–4.

131 Inui S, Fujikawa A, Jitsu M, *et al.* Chest CT Findings in Cases from the Cruise Ship “Diamond Princess” with Coronavirus Disease 2019 (COVID-19). *Radiology: Cardiothoracic Imaging* 2020; **2**: e200110.

132 Isoldi S, Mallardo S, Marcellino A, *et al.* The comprehensive clinic, laboratory, and instrumental evaluation of children with COVID-19: A 6-months prospective study. *J Med Virol* 2021; **93**: 3122–32.

133 Jani S, Jacques SM, Qureshi F, *et al.* Clinical Characteristics of Mother-Infant Dyad and Placental Pathology in COVID-19 Cases in Predominantly African American Population. *AJP Rep* 2021; **11**: e15–20.

134 Jatt LP, Winnett A, Graber CJ, Vallone J, Beenhouwer DO, Goetz MB. Widespread severe acute respiratory coronavirus virus 2 (SARS-CoV-2) laboratory surveillance program to minimize asymptomatic transmission in high-risk inpatient and congregate living settings. *Infect Control Hosp Epidemiol* 2020; : 1–4.

135 Jeong TH, Pak C, Ock M, Lee SH, Son JS, Jeon YJ. Real Asymptomatic SARS-CoV-2 Infection Might Be Rare: Importance of Careful Interviews and Follow-up. *J Korean Med Sci* 2020; **35**: e333.

136 Jiang X-L, Zhang X-L, Zhao X-N, *et al.* Transmission Potential of Asymptomatic and Paucisymptomatic Severe Acute Respiratory Syndrome Coronavirus 2 Infections: A 3-Family Cluster Study in China. *J Infect Dis* 2020; **221**: 1948–52.

137 Jiang Y, Niu W, Wang Q, Zhao H, Meng L, Zhang C. Characteristics of a family cluster of Severe Acute Respiratory Syndrome Coronavirus 2 in Henan, China. J. Infect. 2020; **81**: e46–8.

138 Jiang H, Cheng H, Cao Q, *et al.* Clinical features, laboratory findings and persistence of

virus in 10 children with coronavirus disease 2019 (COVID-19). *Biomed J* 2020; published online Oct. DOI:10.1016/j.bj.2020.10.007.

139 Jones NK, Rivett L, Sparkes D, *et al.* Effective control of SARS-CoV-2 transmission between healthcare workers during a period of diminished community prevalence of COVID-19. *Elife* 2020; **9**. DOI:10.7554/eLife.59391.

140 Jones A, Fialkowski V, Prinzing L, Trites J, Kelso P, Levine M. Assessment of Day-7 Postexposure Testing of Asymptomatic Contacts of COVID-19 Patients to Evaluate Early Release from Quarantine - Vermont, May-November 2020. *MMWR Morb Mortal Wkly Rep* 2021; **70**: 12–3.

141 Joshi RK, Ray RK, Adhya S, Chauhan VPS, Pani S. Spread of COVID-19 by asymptomatic cases: evidence from military quarantine facilities. *BMJ Mil Health* 2020; : bmjmilitary-2020-001669.

142 Liu J-Y, Chen T-J, Hwang S-J. Analysis of community-acquired COVID-19 cases in Taiwan. *J Chin Med Assoc* 2020; **83**: 1087–92.

143 Jung C-Y, Park H, Kim DW, Choi YJ, Kim SW, Chang TI. Clinical Characteristics of Asymptomatic Patients with COVID-19: A Nationwide Cohort Study in South Korea. *Int J Infect Dis* 2020; published online Aug 6. DOI:10.1016/j.ijid.2020.08.001.

144 Just J, Puth M-T, Regenold F, Weckbecker K, Bleckwenn M. Risk factors for a positive SARS-CoV-2 PCR in patients with common cold symptoms in a primary care setting - a retrospective analysis based on a joint documentation standard. *BMC Fam Pract* 2020; **21**: 251.

145 Expert Taskforce for the COVID-19 Cruise Ship Outbreak. Epidemiology of COVID-19 Outbreak on Cruise Ship Quarantined at Yokohama, Japan, February 2020. *Emerg Infect Dis* 2020; **26**. DOI:10.3201/eid2611.201165.

146 Kang Y, You Z, Wang K, *et al.* A retrospective view of pediatric cases infected with SARS-CoV-2 of a middle-sized city in mainland China. *Medicine (Baltimore)* 2020; **99**: e23797.

147 Karout L, Serwat A, El Mais H, Kassab M, Khalid F, Ruiz Mercedes B. COVID-19 Prevalence, Risk Perceptions, and Preventive Behavior in Asymptomatic Latino Population: A Cross-Sectional Study. *Cureus* 2020; **12**: e10707.

148 Kassem AM, Talaat H, Shawky S, *et al.* SARS-CoV-2 infection among healthcare workers of a gastroenterological service in a tertiary care facility. *Arab J Gastroenterol* 2020; published online July 21. DOI:10.1016/j.ajg.2020.07.005.

149 Bai K, Liu W, Liu C, *et al.* Clinical Analysis of 25 COVID-19 Infections in Children. *Pediatr Infect Dis J* 2020; **39**: e100–3.

150 Kennelly SP, Dyer AH, Noonan C, *et al.* Asymptomatic carriage rates and case-fatality of SARS-CoV-2 infection in residents and staff in Irish nursing homes. *Age Ageing* 2020;

published online Sept 28. DOI:10.1093/ageing/afaa220.

151 Khalil A, Hill R, Ladhani S, Pattisson K, O'Brien P. Severe acute respiratory syndrome coronavirus 2 in pregnancy: symptomatic pregnant women are only the tip of the iceberg. *Am J Obstet Gynecol* 2020; **223**: 296–7.

152 Khondaker T, Qader MA, Gosh K, *et al.* Clinical Profile and Outcome of COVID -19 in Children with Pre-Existing Renal Disease. *Int J Pediatr Nephrol* 2021; **9**: 1–6.

153 Ki M, Task Force for 2019-nCoV. Epidemiologic characteristics of early cases with 2019 novel coronavirus (2019-nCoV) disease in Korea. *Epidemiol Health* 2020; **42**: e2020007.

154 Kimball A, Hatfield KM, Arons M, *et al.* Asymptomatic and Presymptomatic SARS-CoV-2 Infections in Residents of a Long-Term Care Skilled Nursing Facility - King County, Washington, March 2020. *MMWR Morb Mortal Wkly Rep* 2020; **69**: 377–81.

155 Kirshblum SC, DeLauter G, Lopreiato MC, *et al.* Screening testing for SARS-CoV -2 upon admission to rehabilitation hospitals in a high COVID -19 prevalence community. *PM&R* 2020; published online July 23. DOI:10.1002/pmrj.12454.

156 Kittang BR, Hofacker S von, Solheim SP, Krüger K, Løland KK, Jansen K. Utbrudd av covid-19 ved tre sykehjem i Bergen. *Tidsskr Sykepl* 2020. DOI:10.4045/tidsskr.20.0405.

157 Koizumi N, Siddique AB, Andalibi A. Assessment of SARS-CoV-2 transmission among attendees of live concert events in Japan using contact-tracing data. *J Travel Med* 2020; **27**. DOI:10.1093/jtm/taaa096.

158 COVID-19 National Emergency Response Center, Epidemiology and Case Management Team, Korea Centers for Disease Control and Prevention. Early Epidemiological and Clinical Characteristics of 28 Cases of Coronavirus Disease in South Korea. *Osong Public Health Res Perspect* 2020; **11**: 8–14.

159 Kristiansen MF, Heimustovu BH, Borg SÁ, *et al.* Epidemiology and Clinical Course of First Wave Coronavirus Disease Cases, Faroe Islands. *Emerg Infect Dis* 2021; **27**: 749–58.

160 Krone M, Noffz A, Richter E, Vogel U, Schwab M. Control of a COVID-19 outbreak in a nursing home by general screening and cohort isolation in Germany, March to May 2020. *Euro Surveill* 2021; **26**. DOI:10.2807/1560-7917.ES.2021.26.1.2001365.

161 Krüger S, Leskien M, Schuller P, *et al.* Performance and feasibility of universal PCR admission screening for SARS-CoV-2 in a German tertiary care hospital. *J Med Virol* 2021; published online Jan 2. DOI:10.1002/jmv.26770.

162 Kute VB, Bhalla AK, Guleria S, *et al.* Clinical profile and outcome of COVID-19 in 250 kidney transplant recipients: A multicenter cohort study from India. *Transplantation* 2020; **Publish Ahead of Print**. DOI:10.1097/TP.0000000000003593.

163 Kute VB, Ray DS, Yadav DK, *et al.* A Multicenter Cohort Study From India of 75 Kidney

Transplants in Recipients Recovered After COVID-19. *Transplantation* 2021; published online March 10. DOI:10.1097/TP.0000000000003740.

164 Kutsuna S, Suzuki T, Hayakawa K, *et al.* SARS-CoV-2 Screening Test for Japanese Returnees From Wuhan, China, January 2020. *Open Forum Infect Dis* 2020; **7**: ofaa243.

165 LaCourse SM, Kachikis A, Blain M, *et al.* Low prevalence of SARS-CoV-2 among pregnant and postpartum patients with universal screening in Seattle, Washington. *Clin Infect Dis* 2020; published online May 30. DOI:10.1093/cid/ciaa675.

166 Ladhani SN, Chow JY, Janarthanan R, *et al.* Investigation of SARS-CoV-2 outbreaks in six care homes in London, April 2020. *EClinicalMedicine* 2020; **26**: 100533.

167 Lai X, Wang M, Qin C, *et al.* Coronavirus Disease 2019 (COVID-2019) Infection Among Health Care Workers and Implications for Prevention Measures in a Tertiary Hospital in Wuhan, China. *JAMA Netw Open* 2020; **3**: e209666.

168 Lamichhane S, Gupta S, Akinjobi G, Ndubuka N. Familial cluster of asymptomatic COVID-19 cases in a First Nation community in Northern Saskatchewan, Canada. *Can Commun Dis Rep* 2021; **47**: 94–6.

169 Lavezzo E, Franchin E, Ciavarella C, *et al.* Suppression of a SARS-CoV-2 outbreak in the Italian municipality of Vo’. *Nature* 2020; published online June 30. DOI:10.1038/s41586-020-2488-1.

170 Thi Quynh Mai Le, Taichiro Takemura, Meng Ling Moi, *et al.* Severe Acute Respiratory Syndrome Coronavirus 2 Shedding by Travelers, Vietnam, 2020. *Emerging Infectious Disease journal* 2020; **26**. DOI:10.3201/eid2607.200591.

171 Lee JY, Hong SW, Hyun M, *et al.* Epidemiological and Clinical Characteristics of Coronavirus Disease 2019 in Daegu, South Korea. *Int J Infect Dis* 2020; published online July 20. DOI:10.1016/j.ijid.2020.07.017.

172 Letizia AG, Ramos I, Obla A, *et al.* SARS-CoV-2 Transmission among Marine Recruits during Quarantine. *N Engl J Med* 2020; **383**: 2407–16.

173 Lewis SS, Smith B, Akinboyo IC, *et al.* Early Experience with Universal Pre-procedural Testing for SARS-CoV-2 in a Relatively Low-Prevalence Area. *Infect Control Hosp Epidemiol* 2020; : 1–9.

174 Li W, Su Y-Y, Zhi S-S, *et al.* Virus shedding dynamics in asymptomatic and mildly symptomatic patients infected with SARS-CoV-2. *Clin Microbiol Infect* 2020; published online July 9. DOI:10.1016/j.cmi.2020.07.008.

175 Li Y, Shi J, Xia J, *et al.* Asymptomatic and Symptomatic Patients With Non-severe Coronavirus Disease (COVID-19) Have Similar Clinical Features and Virological Courses: A Retrospective Single Center Study. *Front Microbiol* 2020; **11**: 1570.

176 Li J, Yang Y, Gong M, *et al.* Aggressive Quarantine Measures Reduce the High Morbidity of COVID-19 in Patients on Maintenance Hemodialysis and Medical Staff of Hemodialysis Facilities in Wuhan, China. *Kidney Dis (Basel)* 2020; **6**: 271–83.

177 Li J, Thoon KC, Chong CY, *et al.* Comparative analysis of symptomatic and asymptomatic SARS-CoV-2 infection in children. *Ann Acad Med Singapore* 2020; **49**: 530–7.

178 Li X, Rong Y, Zhang P, *et al.* Differences in clinical features and laboratory results between adults and children with SARS-CoV-2 infection. *Biomed Res Int* 2020; **2020**: 6342598.

179 Liao J, Fan S, Chen J, *et al.* Epidemiological and Clinical Characteristics of COVID-19 in Adolescents and Young Adults. *Innov J* 2020; **1**: 100001.

180 Lin S, Pan H, Wu H, *et al.* Epidemiological and clinical characteristics of 161 discharged cases with coronavirus disease 2019 in Shanghai, China. *BMC Infect Dis* 2020; **20**: 780.

181 Epidemiology Working Group for NCIP Epidemic Response, Chinese Center for Disease Control and Prevention. [The epidemiological characteristics of an outbreak of 2019 novel coronavirus diseases (COVID-19) in China]. *Zhonghua Liu Xing Bing Xue Za Zhi* 2020; **41**: 145–51.

182 Liu J-Y, Chen T-J, Hwang S-J. Analysis of Imported Cases of COVID-19 in Taiwan: A Nationwide Study. *Int J Environ Res Public Health* 2020; **17**. DOI:10.3390/ijerph17093311.

183 Liu L, Hong X, Su X, *et al.* Optimizing screening strategies for coronavirus disease 2019: A study from Middle China. *J Infect Public Health* 2020; **13**: 868–72.

184 Liu S, Luo H, Wang Y, *et al.* Clinical characteristics and risk factors of patients with severe COVID-19 in Jiangsu province, China: a retrospective multicentre cohort study. *BMC Infect Dis* 2020; **20**: 584.

185 Liu Z, Wu Q, Zou Z, *et al.* Investigation of a family cluster outbreak of COVID-19 indicates the necessity of CT screening for asymptomatic family members in close contact with confirmed patients. *J Thorac Dis* 2020; **12**: 3673–81.

186 Liu J, Huang J, Xiang D. Large SARS-CoV-2 Outbreak Caused by Asymptomatic Traveler, China. *Emerg Infect Dis* 2020; **26**. DOI:10.3201/eid2609.201798.

187 Liu P, Ma Z, Deng Y, *et al.* Characteristics and effectiveness of the Coronavirus disease 2019 (COVID-19) prevention and control in a representative city in China. *Med Sci Monit* 2020; **26**: e927472.

188 Liu P, Niu R, Chen J, *et al.* Epidemiological and clinical features in patients with coronavirus disease 2019 outside of Wuhan, China: Special focus in asymptomatic patients. *PLoS Negl Trop Dis* 2021; **15**: e0009248.

189 Lokken EM, Walker CL, Delaney S, *et al.* Clinical characteristics of 46 pregnant women with a severe acute respiratory syndrome coronavirus 2 infection in Washington State. *Am J*

Obstet Gynecol 2020; published online May 19. DOI:10.1016/j.ajog.2020.05.031.

190 Lombardi A, Consonni D, Carugno M, *et al.* Characteristics of 1573 healthcare workers who underwent nasopharyngeal swab testing for SARS-CoV-2 in Milan, Lombardy, Italy. *Clin Microbiol Infect* 2020; published online June 20. DOI:10.1016/j.cmi.2020.06.013.

191 London V, McLaren R Jr, Atallah F, *et al.* The Relationship between Status at Presentation and Outcomes among Pregnant Women with COVID-19. *Am J Perinatol* 2020; **37**: 991–4.

192 Lopez AS, Hill M, Antezano J, *et al.* Transmission Dynamics of COVID-19 Outbreaks Associated with Child Care Facilities - Salt Lake City, Utah, April-July 2020. *MMWR Morb Mortal Wkly Rep* 2020; **69**: 1319–23.

193 Lu X, Zhang L, Du H, *et al.* SARS-CoV-2 Infection in Children. *N Engl J Med* 2020; **382**: 1663–5.

194 Field Briefing: Diamond Princess COVID-19 Cases, 20 Feb Update. 2020; published online Feb 21. https://www.niid.go.jp/niid/en/2019-ncov-e/9417-covid-dp-fe-02.html (accessed Aug 12, 2020).

195 Luo L, Liu D, Liao X, *et al.* Contact Settings and Risk for Transmission in 3410 Close Contacts of Patients With COVID-19 in Guangzhou, China: A Prospective Cohort Study. *Ann Intern Med* 2020; published online Aug 13. DOI:10.7326/M20-2671.

196 Luo Y, Trevathan E, Qian Z, *et al.* Asymptomatic SARS-CoV-2 Infection in Household Contacts of a Healthcare Provider, Wuhan, China. *Emerg Infect Dis* 2020; **26**: 1930–3.

197 Ly TDA, Zanini D, Laforge V, *et al.* Pattern of SARS-CoV-2 infection among dependant elderly residents living in long-term care facilities in Marseille, France, March-June 2020. *Int J Antimicrob Agents* 2020; **56**: 106219.

198 Lytras T, Dellis G, Flountzi A, *et al.* High prevalence of SARS-CoV-2 infection in repatriation flights to Greece from three European countries. *J Travel Med* 2020; **27**. DOI:10.1093/jtm/taaa054.

199 Ma Y, Xu Q-N, Wang F-L, *et al.* Characteristics of asymptomatic patients with SARS-CoV-2 infection in Jinan, China. *Microbes Infect* 2020; **22**: 212–7.

200 Macartney K, Quinn HE, Pillsbury AJ, *et al.* Transmission of SARS-CoV-2 in Australian educational settings: a prospective cohort study. *Lancet Child Adolesc Health* 2020; published online Aug 3. DOI:10.1016/S2352-4642(20)30251-0.

201 Maechler F, Gertler M, Hermes J, *et al.* Epidemiological and clinical characteristics of SARS-CoV-2 infections at a testing site in Berlin, Germany, March and April 2020 - A cross-sectional study. *Clin Microbiol Infect* 2020; published online Aug 19. DOI:10.1016/j.cmi.2020.08.017.

202 Maggiolo F, Zoboli F, Arosio M, *et al.* SARS-CoV-2 infection in persons living with HIV:

a single center prospective cohort. *J Med Virol* 2020; published online July 24. DOI:10.1002/jmv.26352.

203 Malagón-Rojas JN, Mercado M, Gómez-Rendón CP. SARS-CoV-2 and work-related transmission: results of a prospective cohort of airport workers, 2020. *Rev Bras Med Trab* 2021; **18**: 371–80.

204 Malheiro R, Figueiredo AL, Magalhães JP, *et al.* Effectiveness of contact tracing and quarantine on reducing COVID-19 transmission: a retrospective cohort study. *Public Health* 2020; **189**: 54–9.

205 Maltezou HC, Vorou R, Papadima K, *et al.* Transmission dynamics of SARS-CoV-2 within families with children in Greece: a study of 23 clusters. *J Med Virol* 2020; published online Aug 7. DOI:10.1002/jmv.26394.

206 Maltezou HC, Magaziotou I, Dedoukou X, *et al.* Children and adolescents with SARS-CoV-2 infection: Epidemiology, clinical course and viral loads. *Pediatr Infect Dis J* 2020; **39**: e388–92.

207 Mao S, Huang T, Yuan H, *et al.* Epidemiological analysis of 67 local COVID-19 clusters in Sichuan Province, China. *BMC Public Health* 2020; **20**: 1525.

208 Marcus N, Frizinsky S, Hagin D, *et al.* Minor Clinical Impact of COVID-19 Pandemic on Patients With Primary Immunodeficiency in Israel. *Front Immunol* 2020; **11**: 614086.

209 Marcus JE, Frankel DN, Pawlak MT, *et al.* Risk Factors Associated With COVID-19 Transmission Among US Air Force Trainees in a Congregant Setting. *JAMA Netw Open* 2021; **4**: e210202.

210 Marks M, Millat-Martinez P, Ouchi D, *et al.* Transmission of COVID-19 in 282 clusters in Catalonia, Spain: a cohort study. *Lancet Infect Dis* 2021; **21**: 629–36.

211 Marossy A, Rakowicz S, Bhan A, *et al.* A study of universal SARS-CoV-2 RNA testing of residents and staff in a large group of care homes in South London. *J Infect Dis* 2020; published online Sept 5. DOI:10.1093/infdis/jiaa565.

212 Martin C, Montesinos I, Dauby N, *et al.* Dynamic of SARS-CoV-2 RT-PCR positivity and seroprevalence among high-risk health care workers and hospital staff. *J Hosp Infect* 2020; published online June 25. DOI:10.1016/j.jhin.2020.06.028.

213 Martinez-Fierro ML, Ríos-Jasso J, Garza-Veloz I, *et al.* The role of close contacts of COVID-19 patients in the SARS-CoV-2 transmission: an emphasis on the percentage of non-evaluated positivity in Mexico. *Am J Infect Control* 2020; published online Oct 6. DOI:10.1016/j.ajic.2020.10.002.

214 Martini F, D'Alessio A, Bracchi F, *et al.* On cancer, COVID-19, and CT scans: A monocentric retrospective study. *J Clin Med* 2020; **9**: 3935.

215 Massarotti C, Adriano M, Cagnacci A, *et al.* Asymptomatic SARS-CoV-2 infections in pregnant patients in an Italian city during complete lockdown. *J Med Virol* 2020; published online Aug 25. DOI:10.1002/jmv.26458.

216 Martin-Villares C, Bernal-Sprekelsen M, Molina-Ramirez CP, Bartolome-Benito M, COVID ORL ESP Collaborative Group. Risk of contagion of SARS-CoV-2 among otorhinolaryngologists in Spain during the "Two waves." *Eur Arch Otorhinolaryngol* 2021; published online Jan 19. DOI:10.1007/s00405-020-06582-8.

217 Panda P, Meena M, Singh M, Bairwa M. Non-COVID area of a tertiary care hospital: A major source of nosocomial COVID-19 transmission. *J Family Community Med* 2020; **27**: 212.

218 Melgosa M, Madrid A, Alvárez O, *et al.* SARS-CoV-2 infection in Spanish children with chronic kidney pathologies. *Pediatr Nephrol* 2020; **35**: 1521–4.

219 Menachemi N, Yiannoutsos CT, Dixon BE, *et al.* Population Point Prevalence of SARS-CoV-2 Infection Based on a Statewide Random Sample - Indiana, April 25-29, 2020. *MMWR Morb Mortal Wkly Rep* 2020; **69**: 960–4.

220 Menting T, Krause K, Benz-Tettey F, *et al.* Low-threshold SARS-CoV-2 testing facility for hospital staff: Prevention of COVID-19 outbreaks? *Int J Hyg Environ Health* 2020; **231**: 113653.

221 Merza MA, Haleem Al Mezori AA, Mohammed HM, Abdulah DM. COVID-19 outbreak in Iraqi Kurdistan: The first report characterizing epidemiological, clinical, laboratory, and radiological findings of the disease. *Diabetes Metab Syndr* 2020; **14**: 547–54.

222 Meyers KJ, Jones ME, Goetz IA, *et al.* A cross-sectional community-based observational study of asymptomatic SARS-CoV-2 prevalence in the greater Indianapolis area. *J Med Virol* 2020; published online June 16. DOI:10.1002/jmv.26182.

223 Meyers KJ, Dillman B, Williams C, *et al.* Follow-up of SARS-CoV-2 positive subgroup from the Asymptomatic novel CORonavirus iNFection study. *J Med Virol* 2021; published online Jan 19. DOI:10.1002/jmv.26810.

224 Migisha R, Kwesiga B, Mirembe BB, *et al.* Early cases of SARS-CoV-2 infection in Uganda: epidemiology and lessons learned from risk-based testing approaches - March-April 2020. *Global Health* 2020; **16**: 114.

225 Miller ES, Grobman WA, Sakowicz A, Rosati J, Peaceman AM. Clinical Implications of Universal Severe Acute Respiratory Syndrome Coronavirus 2 (SARS-CoV-2) Testing in Pregnancy. *Obstet Gynecol* 2020; **136**: 232–4.

226 Min Y, Cheng L, Tu C, *et al.* Clinical characteristics of deceased hemodialysis patients affected by COVID-19. *Int Urol Nephrol* 2021; published online Jan 2. DOI:10.1007/s11255-020-02700-x.

227 Miyahara R, Tsuchiya N, Yasuda I, *et al.* Familial Clusters of Coronavirus Disease in 10 Prefectures, Japan, February−May 2020. *Emerging Infectious Disease journal* 2021; **27**: 915.

228 Mostafa A, Kandil S, El-Sayed MH, *et al.* Universal COVID-19 screening of 4040 health care workers in a resource-limited setting: an Egyptian pilot model in a university with 12 public hospitals and medical centers. *Int J Epidemiol* 2020; published online Oct 23. DOI:10.1093/ije/dyaa173.

229 Myles PS, Wallace S, Story DA, *et al.* COVID-19 risk in elective surgery during a second wave: a prospective cohort study. *ANZ J Surg* 2021; **91**: 22–6.

230 Nakajo K, Nishiura H. Transmissibility of asymptomatic COVID-19: Data from Japanese clusters. *Int J Infect Dis* 2021; **105**: 236–8.

231 Namal E. Single center experience on screening oncology patients for covid-19 before anti-cancer treatment. *Int J Hematol Oncol* 2020; **30**: 207–12.

232 de Miguel Negro M, González Tallada A, de Nadal M, *et al.* Pre-operative prevalence of asymptomatic carriers of COVID-19 in hospitals in Catalonia during the first wave after the resumption of surgical activity. *Cir Esp* 2021; published online Jan 29. DOI:10.1016/j.ciresp.2021.01.014.

233 Ng O-T, Marimuthu K, Chia P-Y, *et al.* SARS-CoV-2 Infection among Travelers Returning from Wuhan, China. *N Engl J Med* 2020; **382**: 1476–8.

234 Nicolás D, Camós-Carreras A, Spencer F, *et al.* A Prospective Cohort of SARS-CoV-2-Infected Health Care Workers: Clinical Characteristics, Outcomes, and Follow-up Strategy. *Open Forum Infect Dis* 2021; **8**: ofaa592.

235 Nishiura H, Kobayashi T, Yang Y, *et al.* The Rate of Underascertainment of Novel Coronavirus (2019-nCoV) Infection: Estimation Using Japanese Passengers Data on Evacuation Flights. *J Clin Med Res* 2020; **9**. DOI:10.3390/jcm9020419.

236 Njuguna H, Wallace M, Simonson S, *et al.* Serial Laboratory Testing for SARS-CoV-2 Infection Among Incarcerated and Detained Persons in a Correctional and Detention Facility - Louisiana, April-May 2020. *MMWR Morb Mortal Wkly Rep* 2020; **69**: 836–40.

237 Norsa L, Cosimo P, Indriolo A, Sansotta N, D'Antiga L, Callegaro A. Asymptomatic SARS-CoV-2 infection in patients with inflammatory bowel disease under biologic treatment. *Gastroenterology* 2020; published online Aug 26. DOI:10.1053/j.gastro.2020.08.046.

238 Oduro-Mensah E, Tetteh J, Adomako I, Adjei-Mensah E, Yeboah A. Clinical Features of COVID-19 in Ghana: Symptomatology, Illness severity and Co-morbid Non-communicable diseases. *Ghana Med J* 2020; **54**. DOI:10.4314/gmj.v54i4s.5.

239 Olmos C, Campaña G, Monreal V, *et al.* SARS-CoV-2 infection in asymptomatic

healthcare workers at a clinic in Chile. *PLoS One* 2021; **16**: e0245913.

240 Ornaghi S, Callegari C, Milazzo R, *et al.* Performance of an extended triage questionnaire to detect suspected cases of Severe Acute Respiratory Syndrome Coronavirus 2 (SARS-CoV-2) infection in obstetric patients: Experience from two large teaching hospitals in Lombardy, Northern Italy. *PLoS One* 2020; **15**: e0239173.

241 Ossareh S, Bagheri M, Abbasi M, Abolfathi S, Bohlooli A. Role of Screening for COVID-19 in Hemodialysis Wards, Results of a Single Center Study. *Iran J Kidney Dis* 2020; **14**: 389–98.

242 Oster Y, Wolf DG, Olshtain-Pops K, Rotstein Z, Schwartz C, Benenson S. Proactive screening approach for SARS-CoV-2 among healthcare workers. *Clin Microbiol Infect* 2020; published online Aug 18. DOI:10.1016/j.cmi.2020.08.009.

243 Panagiotakopoulos L, Myers TR, Gee J, *et al.* SARS-CoV-2 Infection Among Hospitalized Pregnant Women: Reasons for Admission and Pregnancy Characteristics - Eight U.S. Health Care Centers, March 1-May 30, 2020. *MMWR Morb Mortal Wkly Rep* 2020; **69**: 1355–9.

244 Park SY, Kim Y-M, Yi S, *et al.* Coronavirus Disease Outbreak in Call Center, South Korea. *Emerg Infect Dis* 2020; **26**: 1666–70.

245 Park JH, Jang JH, Lee K, Yoo SJ, Shin H. COVID-19 outbreak and presymptomatic transmission in pilgrim travelers who returned to Korea from Israel. *J Korean Med Sci* 2020; **35**: e424.

246 Parri N, Magistà AM, Marchetti F, *et al.* Characteristic of COVID-19 infection in pediatric patients: early findings from two Italian Pediatric Research Networks. *Eur J Pediatr* 2020; **179**: 1315–23.

247 Parri N, Lenge M, Cantoni B, *et al.* COVID-19 in 17 Italian pediatric emergency departments. *Pediatrics* 2020; **146**: e20201235.

248 Passarelli VC, Faico-Filho K, Moreira LVL, *et al.* Asymptomatic SARS-CoV-2 infections in hospitalized patients. *Infect Control Hosp Epidemiol* 2020; : 1–9.

249 Passarelli VC, Faico-Filho K, Moreira LVL, *et al.* Asymptomatic COVID-19 in hospital visitors: The underestimated potential of viral shedding. *Int J Infect Dis* 2020; **102**: 412–4.

250 Patberg ET, Adams T, Rekawek P, *et al.* Coronavirus disease 2019 infection and placental histopathology in women delivering at term. *Am J Obstet Gynecol* 2020; published online Oct. DOI:10.1016/j.ajog.2020.10.020.

251 Patel MC, Chaisson LH, Borgetti S, *et al.* Asymptomatic SARS-CoV-2 infection and COVID-19 mortality during an outbreak investigation in a skilled nursing facility. *Clin Infect Dis* 2020; published online June 16. DOI:10.1093/cid/ciaa763.

252 Patel AB, Clifford A, Creaden J, *et al.* SARS-CoV-2 Point Prevalence among Asymptomatic Hospitalized Children and Subsequent Healthcare Worker Evaluation. *J Pediatric Infect Dis Soc* 2020; published online Aug 28. DOI:10.1093/jpids/piaa102.

253 Patil UP, Krishnan P, Abudinen-Vasquez S, Maru S, Noble L. Severe Acute Respiratory Syndrome Coronavirus 2 (SARS-CoV-2) Positive Newborns of COVID-19 Mothers After Dyad-Care: A Case Series. *Cureus* 2021; **13**: e12528.

254 Pavli A, Smeti P, Papadima K, *et al.* A cluster of COVID-19 in pilgrims to Israel. *J Travel Med* 2020; published online June 25. DOI:10.1093/jtm/taaa102.

255 Pavli A, Smeti P, Hadjianastasiou S, *et al.* In-flight transmission of COVID-19 on flights to Greece: An epidemiological analysis. *Travel Med Infect Dis* 2020; **38**: 101882.

256 Payne DC, Smith-Jeffcoat SE, Nowak G, *et al.* SARS-CoV-2 Infections and Serologic Responses from a Sample of U.S. Navy Service Members - USS Theodore Roosevelt, April 2020. *MMWR Morb Mortal Wkly Rep* 2020; **69**: 714–21.

257 Petersen I, Phillips A. Three quarters of people with SARS-CoV-2 infection are asymptomatic: Analysis of English household survey data. *Clin Epidemiol* 2020; **12**: 1039–43.

258 Pham QT, Rabaa MA, Duong HL, *et al.* The first 100 days of SARS-CoV-2 control in Vietnam. *Clin Infect Dis* 2020; published online Aug 1. DOI:10.1093/cid/ciaa1130.

259 Pirnay J-P, Selhorst P, Cochez C, *et al.* Study of a SARS-CoV-2 Outbreak in a Belgian Military Education and Training Center in Maradi, Niger. *Viruses* 2020; **12**. DOI:10.3390/v12090949.

260 Plucinski MM, Wallace M, Uehara A, *et al.* COVID-19 in Americans aboard the Diamond Princess cruise ship. *Clin Infect Dis* 2020; published online Aug 12. DOI:10.1093/cid/ciaa1180.

261 Pollán M, Pérez-Gómez B, Pastor-Barriuso R, *et al.* Prevalence of SARS-CoV-2 in Spain (ENE-COVID): a nationwide, population-based seroepidemiological study. *Lancet* 2020; published online July 3. DOI:10.1016/S0140-6736(20)31483-5.

262 Pongpirul WA, Mott JA, Woodring JV, *et al.* Clinical Characteristics of Patients Hospitalized with Coronavirus Disease, Thailand. *Emerg Infect Dis* 2020; **26**: 1580–5.

263 Prabhu M, Cagino K, Matthews KC, *et al.* Pregnancy and postpartum outcomes in a universally tested population for SARS-CoV-2 in New York City: A prospective cohort study. *BJOG* 2020; published online July 7. DOI:10.1111/1471-0528.16403.

264 Pray IW, Gibbons-Burgener SN, Rosenberg AZ, *et al.* COVID-19 outbreak at an overnight summer school retreat - Wisconsin, July-August 2020. *MMWR Morb Mortal Wkly Rep* 2020; **69**: 1600–4.

265 Puylaert CAJ, Scheijmans JCG, Borgstein ABJ, *et al.* Yield of Screening for COVID-19 in Asymptomatic Patients Prior to Elective or Emergency Surgery Using Chest CT and RT-PCR (SCOUT): Multicenter Study. *Ann Surg* 2020; **Publish Ahead of Print**. DOI:10.1097/SLA.0000000000004218.

266 Qian G, Yang N, Ma AHY, *et al.* COVID-19 Transmission Within a Family Cluster by Presymptomatic Carriers in China. *Clin Infect Dis* 2020; **71**: 861–2.

267 Xingqiang P, Yi C, Aihong W, *et al.* Study on transmission dynamic of 15 clusters of coronavirus disease 2019 cases in Ningbo. *Chinese Journal of Epidemiology* 2020; **41**: E066–E066.

268 Qin W, Sun J, Xu P, *et al.* The descriptive epidemiology of coronavirus disease 2019 during the epidemic period in Lu'an, China: achieving limited community transmission using proactive response strategies. *Epidemiol Infect* 2020; **148**: e132.

269 Qiu H, Wu J, Hong L, Luo Y, Song Q, Chen D. Clinical and epidemiological features of 36 children with coronavirus disease 2019 (COVID-19) in Zhejiang, China: an observational cohort study. *Lancet Infect Dis* 2020; **20**: 689–96.

270 Qiu C, Deng Z, Xiao Q, *et al.* Transmission and clinical characteristics of coronavirus disease 2019 in 104 outside-Wuhan patients, China. *J Med Virol* 2020; published online May 5. DOI:10.1002/jmv.25975.

271 Rajme-López S, González-Lara MF, Ortiz-Brizuela E, *et al.* Large-scale screening for severe acute respiratory coronavirus virus 2 (SARS-CoV-2) among healthcare workers: Prevalence and risk factors for asymptomatic and pauci-symptomatic carriers, with emphasis on the use of personal protective equipment (PPE). *Infect Control Hosp Epidemiol* 2021; : 1–5.

272 Ralli M, Morrone A, Arcangeli A, Ercoli L. Asymptomatic patients as a source of transmission of COVID-19 in homeless shelters. *Int J Infect Dis* 2020; **103**: 243–5.

273 Randremanana RV, Andriamandimby S-F, Rakotondramanga JM, *et al.* The COVID-19 epidemic in Madagascar: clinical description and laboratory results of the first wave, march-september 2020. *Influenza Other Respi Viruses* 2021; published online Feb 15. DOI:10.1111/irv.12845.

274 Reale ML, Bironzo P, Bertaglia V, *et al.* SARS-CoV-2 Infection in Cancer Patients: A Picture of an Italian Onco-Covid Unit. 2020; published online Aug 19. DOI:10.3389/fonc.2020.01722.

275 Reale SC, Lumbreras-Marquez MI, King CH, *et al.* Patient characteristics associated with SARS-CoV-2 infection in parturients admitted for labour and delivery in Massachusetts during the spring 2020 surge: A prospective cohort study. *Paediatr Perinat Epidemiol* 2021; **35**: 24–33.

276 Reid RJ, Rosella L, Milijasevic N, Small LN. Mass testing for asymptomatic COVID-19

infection among health care workers at a large Canadian hospital. *Official Journal of the Association of Medical Microbiology and Infectious Disease Canada* 2020; **5**: 245–50.

277 Ren R, Zhang Y, Li Q, *et al.* Asymptomatic SARS-CoV-2 Infections Among Persons Entering China From April 16 to October 12, 2020. *JAMA* 2021; **325**: 489–92.

278 Rincón A, Moreso F, López-Herradón A, *et al.* The keys to control a COVID-19 outbreak in a haemodialysis unit. *Clin Kidney J* 2020; **13**: 542–9.

279 Rivera F, Safdar N, Ledeboer N, Schaack G, Chen DJ, Munoz-Price LS. Prevalence of SARS-CoV-2 asymptomatic infections in two large academic health systems in Wisconsin. *Clin Infect Dis* 2020; published online Aug 19. DOI:10.1093/cid/ciaa1225.

280 Rivett L, Sridhar S, Sparkes D, *et al.* Screening of healthcare workers for SARS-CoV-2 highlights the role of asymptomatic carriage in COVID-19 transmission. *Elife* 2020; **9**. DOI:10.7554/eLife.58728.

281 Roberts SC, Peaper DR, Thorne CD, *et al.* Mass severe acute respiratory coronavirus virus 2 (SARS-CoV-2) testing of asymptomatic healthcare personnel. *Infect Control Hosp Epidemiol* 2021; : 1–2.

282 Rogers JH, Link AC, McCulloch D, *et al.* Characteristics of COVID-19 in Homeless Shelters : A Community-Based Surveillance Study. *Ann Intern Med* 2020; published online Sept 15. DOI:10.7326/M20-3799.

283 Roxby AC, Greninger AL, Hatfield KM, *et al.* Outbreak Investigation of COVID-19 Among Residents and Staff of an Independent and Assisted Living Community for Older Adults in Seattle, Washington. *JAMA Intern Med* 2020; published online May 21. DOI:10.1001/jamainternmed.2020.2233.

284 Sabetian G, Moghadami M, Hashemizadeh Fard Haghighi L, *et al.* COVID-19 infection among healthcare workers: a cross-sectional study in southwest Iran. *Virol J* 2021; **18**: 58.

285 Sacco G, Foucault G, Briere O, Annweiler C. COVID-19 in seniors: Findings and lessons from mass screening in a nursing home. *Maturitas* 2020; **141**: 46–52.

286 Saeed K, Pelosi E, Mahobia N, *et al.* Investigations, actions and learning from an outbreak of SARS-CoV-2 infection among healthcare workers in the United Kingdom. *J Infect Prev* 2020; : 175717742097679.

287 Huerta Saenz IH, Elías Estrada JC, Campos Del Castillo K, Muñoz Taya R, Coronado JC. Características materno perinatales de gestantes COVID-19 en un hospital nacional de Lima, Perú. *Rev peru ginecol obstet* 2020; **66**. DOI:10.31403/rpgo.v66i2245.

288 Ossami Saidy RR, Globke B, Pratschke J, Schoening W, Eurich D. Successful implementation of preventive measures leads to low relevance of SARS-CoV-2 in liver transplant patients: Observations from a German outpatient department. *Transpl Infect Dis* 2020; : e13363.

289 Sakowicz A, Ayala AE, Ukeje CC, Witting CS, Grobman WA, Miller ES. Risk Factors for SARS-CoV2 Infection in Pregnant Women. *Am J Obstet Gynecol MFM* 2020; : 100198.

290 Saluja M, Pillai D, Jeliya S, Bauddh N, Chandel R. COVID 19- Clinical Profile, Radiological Presentation, Prognostic Predictors, Complications and Outcome: A Perspective from the Indian Subcontinent. *J Assoc Physicians India* 2020; **68**: 13–8.

291 Samrah SM, W Al-Mistarehi A-H, Ibnian AM, *et al.* COVID-19 outbreak in Jordan: Epidemiological features, clinical characteristics, and laboratory findings. *Ann Med Surg (Lond)* 2020; **57**: 103–8.

292 Sanchez GV, Biedron C, Fink LR, *et al.* Initial and Repeated Point Prevalence Surveys to Inform SARS-CoV-2 Infection Prevention in 26 Skilled Nursing Facilities - Detroit, Michigan, March-May 2020. *MMWR Morb Mortal Wkly Rep* 2020; **69**: 882–6.

293 Sastry SR, Pryor R, Raybould JE, *et al.* Universal screening for the SARS-CoV-2 virus on hospital admission in an area with low COVID-19 prevalence. *Infect Control Hosp Epidemiol* 2020; **41**: 1231–3.

294 Saurabh S, Kumar R, Kumar N, *et al.* Dynamics of SARS-CoV-2 transmission among Indian nationals evacuated from Iran. *Disaster Med Public Health Prep* 2020; : 1–7.

295 Savirón-Cornudella R, Villalba A, Zapardiel J, Andeyro-Garcia M, Esteban LM, Pérez-López FR. Severe acute respiratory syndrome coronavirus 2 (SARS-CoV-2) universal screening in gravids during labor and delivery. *Eur J Obstet Gynecol Reprod Biol* 2020; **256**: 400–4.

296 Savirón-Cornudella R, Villalba A, Esteban LM, *et al.* Screening of severe acute respiratory syndrome coronavirus-2 infection during labor and delivery using polymerase chain reaction and immunoglobulin testing. *Life Sci* 2021; **271**: 119200.

297 Scheier T, Schibli A, Eich G, *et al.* Universal Admission Screening for SARS-CoV-2 Infections among Hospitalized Patients, Switzerland, 2020. *Emerg Infect Dis* 2021; **27**: 404–10.

298 Schwierzeck V, König JC, Kühn J, *et al.* First reported nosocomial outbreak of severe acute respiratory syndrome coronavirus 2 (SARS-CoV-2) in a pediatric dialysis unit. *Clin Infect Dis* 2020; published online April 27. DOI:10.1093/cid/ciaa491.

299 See KC, Liew SM, Ng DCE, *et al.* COVID-19: Four Paediatric Cases in Malaysia. *Int J Infect Dis* 2020; **94**: 125–7.

300 Senanayake AP, Indrakantha D, Sumathipala S, *et al.* Features of Covid-19 patients detected during community screening: A study from a rural hospital in Sri Lanka. *Ceylon Med J* 2020; **65**: 67.

301 Setiawaty V, Kosasih H, Mardian Y, *et al.* The identification of first COVID-19 cluster in Indonesia. *Am J Trop Med Hyg* 2020; **103**: 2339–42.

302 Sharma AK, Chapagain RH, Bista KP, *et al.* Epidemiological and clinical profile of COVID-19 in Nepali children: An initial experience. *J Nepal Paediatr Soc* 2020; **40**: 202–9.

303 Sharma N, Seehra N, Kabra S. Pregnancy with covid-19 infection and fetomaternal outcomes. *J evol med dent sci* 2021; **10**: 23–7.

304 Sharma R, Seth S, Sharma R, Yadav S, Mishra P, Mukhopadhyay S. Perinatal outcome and possible vertical transmission of coronavirus disease 2019: experience from North India. *Clin Exp Pediatr* 2021; **64**: 239–46.

305 Shen Q, Guo W, Guo T, *et al.* Novel coronavirus infection in children outside of Wuhan, China. *Pediatr Pulmonol* 2020; **55**: 1424–9.

306 Shen J, Sun J, Zhao D, *et al.* Characteristics of nosocomial infections in children screened for SARS-CoV-2 infection in China. *Med Sci Monit* 2020; **26**: e928835.

307 Shi SM, Bakaev I, Chen H, Travison TG, Berry SD. Risk Factors, Presentation, and Course of COVID-19 in a Large, Academic Long-term Care Facility. *J Am Med Dir Assoc* 2020; published online Aug. DOI:10.1016/j.jamda.2020.08.027.

308 Shi L, Li Q, Li K, *et al.* Quarantine at home may not enough!-from the epidemiological data in Shaanxi Province of China. *BMC Res Notes* 2020; **13**: 506.

309 Shmakov RG, Prikhodko A, Polushkina E, *et al.* Clinical course of novel COVID-19 infection in pregnant women. *J Matern Fetal Neonatal Med* 2020; : 1–7.

310 Singer JS, Cheng EM, Murad D, *et al.* Low Prevalence (0.13%) of COVID-19 Infection in Asymptomatic Pre-operative/Pre-procedure Patients at a Large Academic Medical Center Informs Approaches to Perioperative Care. *Surgery* 2020; published online Aug 14. DOI:10.1016/j.surg.2020.07.048.

311 Singh V, Choudhary A, Datta MR, Ray A. Maternal and Neonatal Outcomes of COVID-19 in Pregnancy: A Single-Centre Observational Study. *Cureus* 2021; **13**: e13184.

312 Son H, Lee H, Lee M, *et al.* Epidemiological characteristics of and containment measures for COVID-19 in Busan, Korea. *Epidemiol Health* 2020; **42**: e2020035.

313 Song W, Li J, Zou N, Guan W, Pan J, Xu W. Clinical features of pediatric patients with coronavirus disease (COVID-19). *J Clin Virol* 2020; **127**: 104377.

314 Soriano-Arandes A, Gatell A, Serrano P, *et al.* Household SARS-CoV-2 transmission and children: a network prospective study. *Clin Infect Dis* 2021; published online March 12. DOI:10.1093/cid/ciab228.

315 Soysal A, Gönüllü E, Arslan H, *et al.* Comparison of clinical and laboratory features and treatment options of 237 Comparison of clinical and laboratory features and treatment options of 237 symptomatic and asymptomatic children infected with SARS-CoV-2 in the early phase of the COVID-19 pandemic in Turkey. *Jpn J Infect Dis* 2020; published online

Nov 30. DOI:10.7883/yoken.JJID.2020.781.

316 Stadler RN, Maurer L, Aguilar-Bultet L, *et al.* Systematic screening on admission for SARS-CoV-2 to detect asymptomatic infections. *Antimicrob Resist Infect Control* 2021; **10**: 44.

317 Stessel B, Callebaut I, Polus F, *et al.* Evaluation of a comprehensive pre-procedural screening protocol for COVID-19 in times of a high SARS CoV-2 prevalence: a prospective cross-sectional study. *Ann Med* 2021; **53**: 337–44.

318 Stock AD, Bader ER, Cezayirli P, *et al.* COVID-19 Infection Among Healthcare Workers: Serological Findings Supporting Routine Testing. *Front Med* 2020; **7**: 471.

319 Sugano N, Ando W, Fukushima W. Cluster of SARS-CoV-2 infections linked to music clubs in Osaka, Japan: asymptomatically infected persons can transmit the virus as soon as 2 days after infection. *J Infect Dis* 2020; published online Aug 25. DOI:10.1093/infdis/jiaa542.

320 Suh HJ, Kim DH, Heo EY, *et al.* Clinical Characteristics of COVID-19: Clinical Dynamics of Mild Severe Acute Respiratory Syndrome Coronavirus 2 Infection Detected by Early Active Surveillance. *J Korean Med Sci* 2020; **35**: e297.

321 Sun D, Zhu F, Wang C, *et al.* Children Infected With SARS-CoV-2 From Family Clusters. *Front Pediatr* 2020; **8**: 386.

322 Sutton D, Fuchs K, D'Alton M, Goffman D. Universal Screening for SARS-CoV-2 in Women Admitted for Delivery. *N Engl J Med* 2020; **382**: 2163–4.

323 Szegedi S, Huf W, Miháltz K, Vécsei-Marlovits PV. Prevalence of SARS-CoV-2 infection in patients presenting for intravitreal injection. *Spektrum Augenheilkd* 2020; : 1–5.

324 Tabata S, Imai K, Kawano S, *et al.* Clinical characteristics of COVID-19 in 104 people with SARS-CoV-2 infection on the Diamond Princess cruise ship: a retrospective analysis. *Lancet Infect Dis* 2020; published online June 12. DOI:10.1016/S1473-3099(20)30482-5.

325 Tambe MP, Parande MA, Tapare VS, *et al.* An epidemiological study of laboratory confirmed COVID-19 cases admitted in a tertiary care hospital of Pune, Maharashtra. *Indian J Public Health* 2020; **64**: S183–7.

326 Tan-Loh J, Cheong BMK. A descriptive analysis of clinical characteristics of COVID-19 among healthcare workers in a district specialist hospital. *Med J Malaysia* 2021; **76**: 24–8.

327 TAN Xin, HUANG Juan, ZHAO Fen, ZHOU Yan, LI Jie-Qiong, WANG Xiang-Yun. 长沙市儿童新型冠状病毒感染13例临床特征分析. 中国当代儿科杂志; **22**: 294–8.

328 Tan Y-P, Tan B-Y, Pan J, Wu J, Zeng S-Z, Wei H-Y. Epidemiologic and clinical characteristics of 10 children with coronavirus disease 2019 in Changsha, China. *J Clin Virol* 2020; **127**: 104353.

329 Tan JK, Leong D, Munusamy H, *et al.* The prevalence and clinical significance of Presymptomatic COVID-19 patients: how we can be one step ahead in mitigating a deadly pandemic. *BMC Infect Dis* 2021; **21**: 249.

330 Tang O, Bigelow BF, Sheikh F, *et al.* Outcomes of nursing home COVID-19 patients by initial symptoms and comorbidity: Results of universal testing of 1970 residents. *J Am Med Dir Assoc* 2020; **21**: 1767-1773.e1.

331 Ali T, Al-Ali A, Fajji L, *et al.* Coronavirus Disease-19: Disease Severity and Outcomes of Solid Organ Transplant Recipients: Different Spectrum of Disease in Different Populations? *Transplantation* 2020; published online Aug 24. DOI:10.1097/TP.0000000000003433.

332 Tawar S, Diva Reddy G, Ray S, Chawla N, Garg S. Rapid response and mitigation measures in control of COVID-19 cases in an industrial warehouse of Western Maharashtra, India. *Journal of Marine Medical Society* 2020; **22**: 220.

333 Taylor J, Carter RJ, Lehnertz N, *et al.* Serial Testing for SARS-CoV-2 and Virus Whole Genome Sequencing Inform Infection Risk at Two Skilled Nursing Facilities with COVID-19 Outbreaks - Minnesota, April-June 2020. *MMWR Morb Mortal Wkly Rep* 2020; **69**: 1288–95.

334 Temel H, Gündüz M, Arslan H, *et al.* Evaluation of the clinical features of 81 patients with COVID-19: An unpredictable disease in children. *J Pediatr Infect Dis* 2020; published online Dec 16. DOI:10.1055/s-0040-1721511.

335 Vivian Thangaraj JW, Murhekar M, Mehta Y, *et al.* A cluster of SARS-CoV-2 infection among Italian tourists visiting India, March 2020. *Indian J Med Res* 2020; **151**: 438–43.

336 Thiel SL, Weber MC, Risch L, *et al.* Flattening the curve in 52 days: characterisation of the COVID-19 pandemic in the Principality of Liechtenstein - an observational study. *Swiss Med Wkly* 2020; **150**: w20361.

337 Thompson JW Jr, Mikolajewski AJ, Kissinger P, *et al.* An Epidemiologic Study of COVID-19 Patients in a State Psychiatric Hospital: High Penetrance With Early CDC Guidelines. *Psychiatr Serv* 2020; : appips202000270.

338 Tian S, Hu N, Lou J, *et al.* Characteristics of COVID-19 infection in Beijing. *J Infect* 2020; **80**: 401–6.

339 Tompkins LK, Gunn JKL, Cherney B, *et al.* Mass SARS-CoV-2 Testing in a Dormitory-Style Correctional Facility in Arkansas. *American Journal of Public Health* 2021; **111**: 907–16.

340 Tong Z-D, Tang A, Li K-F, *et al.* Potential Presymptomatic Transmission of SARS-CoV-2, Zhejiang Province, China, 2020. *Emerg Infect Dis* 2020; **26**: 1052–4.

341 Trahan M-J, Mitric C, Malhamé I, Abenhaim HA. Screening and testing pregnant patients for SARS-CoV-2: First-wave experience of a designated COVID-19 hospitalization centre

in Montreal. *J Obstet Gynaecol Can* 2020; published online Nov 5. DOI:10.1016/j.jogc.2020.11.001.

342 Tsou T-P, Chen W-C, Huang AS-E, Chang S-C, Taiwan COVID-19 Outbreak Investigation Team. Epidemiology of the first 100 cases of COVID-19 in Taiwan and its implications on outbreak control. *J Formos Med Assoc* 2020; published online July 13. DOI:10.1016/j.jfma.2020.07.015.

343 Venkataram T, Goyal N, Kalita D, *et al.* Deployment of neurosurgeons at the warfront against Coronavirus disease of 2019 (COVID-19). *World Neurosurg* 2020; **144**: e561–7.

344 Villa J, Pannu T, McWilliams C, *et al.* Results of preoperative screening for COVID-19 correlate with the incidence of infection in the general population -a tertiary care experience. *Hosp Pract* 2021; : 1–5.

345 Wadhwa A, Fisher KA, Silver R, *et al.* Identification of presymptomatic and asymptomatic cases using cohort-based testing approaches at a large correctional facility - Chicago, Illinois, USA, May 2020. *Clin Infect Dis* 2020; published online Dec 3. DOI:10.1093/cid/ciaa1802.

346 Wang Y, Tong J, Qin Y, *et al.* Characterization of an asymptomatic cohort of SARS-COV-2 infected individuals outside of Wuhan, China. *Clin Infect Dis* 2020; published online May 22. DOI:10.1093/cid/ciaa629.

347 Wang G, Guan J-L, Zhu X-Q, *et al.* Infection, screening, and psychological stress of health care workers with COVID-19 in a non-frontline clinical department. *Disaster Med Public Health Prep* 2020; : 1–25.

348 Wang M, Nie X, Huang S, *et al.* Epidemiological characteristics and transmission dynamics of paediatric cases with coronavirus disease 2019 in Hubei province, China. *J Paediatr Child Health* 2020; published online Dec 8. DOI:10.1111/jpc.15287.

349 Ling W, Jing S, Lingling M, *et al.* Source investigation on a familiar cluster of coronavirus disease 2019 in Dandong city of Liaoning Province. *Chinese Journal of Preventive Medicine* 2021; **55**: 120–2.

350 Wanwan S, Feng L, Jinren P, *et al.* Epidemiological characteristics of 2019 novel coronavirus family clustering in Zhejiang Province. *Chinese Journal of Preventive Medicine* 2020; **54**: E027–E027.

351 Wi YM, Lim SJ, Kim S-H, *et al.* Response System for and Epidemiological Features of COVID-19 in Gyeongsangnam-do Province in South Korea. *Clin Infect Dis* 2020; published online July 16. DOI:10.1093/cid/ciaa967.

352 Wong J, Abdul Aziz ABZ, Chaw L, *et al.* High proportion of asymptomatic and presymptomatic COVID-19 infections in travelers and returning residents to Brunei. *J Travel Med* 2020; published online May 5. DOI:10.1093/jtm/taaa066.

353 Wong J, Jamaludin SA, Alikhan MF, Chaw L. Asymptomatic transmission of SARS-CoV-2 and implications for mass gatherings. *Influenza Other Respi Viruses* 2020; published online May 30. DOI:10.1111/irv.12767.

354 Wu J, Huang Y, Tu C, *et al.* Household Transmission of SARS-CoV-2, Zhuhai, China, 2020. *Clin Infect Dis* 2020; published online May 11. DOI:10.1093/cid/ciaa557.

355 吴华平, Hua-Ping WU, 李兵飞, *et al.* 江西地区23例18岁以下儿童2019冠状病毒病临床分析. 2020; published online May 15. DOI:10.7499/j.issn.1008-8830.2003202.

356 Wu S, Xue L, Legido-Quigley H, *et al.* Understanding factors influencing the length of hospital stay among non-severe COVID-19 patients: A retrospective cohort study in a Fangcang shelter hospital. *PLoS One* 2020; **15**: e0240959.

357 Xi A, Zhuo M, Dai J, *et al.* Epidemiological and clinical characteristics of discharged patients infected with SARS-CoV-2 on the Qinghai Plateau. *J Med Virol* 2020; published online May 21. DOI:10.1002/jmv.26032.

358 Xie W, Chen Z, Wang Q, *et al.* Infection and disease spectrum in individuals with household exposure to SARS-CoV-2: A family cluster cohort study. *J Med Virol* 2021; **93**: 3033–46.

359 Xiong F, Tang H, Liu L, *et al.* Clinical Characteristics of and Medical Interventions for COVID-19 in Hemodialysis Patients in Wuhan, China. *J Am Soc Nephrol* 2020; **31**: 1387–97.

360 Xu H, Liu E, Xie J, *et al.* A follow-up study of children infected with SARS-CoV-2 from Western China. Infectious Diseases (except HIV/AIDS). 2020; published online April 24. DOI:10.1101/2020.04.20.20073288.

361 Xu X, Nie S, Sun J, *et al.* The Cumulative Rate of SARS-CoV-2 Infection in Chinese Hemodialysis Patients. *Kidney International Reports* 2020; published online July 18. DOI:10.1016/j.ekir.2020.07.010.

362 Yan J, Guo J, Fan C, *et al.* Coronavirus disease 2019 in pregnant women: a report based on 116 cases. *Am J Obstet Gynecol* 2020; **223**: 111.e1-111.e14.

363 Yang M-C, Hung P-P, Wu Y-K, Peng M-Y, Chao Y-C, Su W-L. A three-generation family cluster with COVID-19 infection: should quarantine be prolonged? *Public Health* 2020; **185**: 31–3.

364 Yang R, Gui X, Xiong Y. Comparison of Clinical Characteristics of Patients with Asymptomatic vs Symptomatic Coronavirus Disease 2019 in Wuhan, China. *JAMA Netw Open* 2020; **3**: e2010182.

365 Yang J, Zhao X, Liu X, *et al.* Clinical Characteristics and Eosinophils in Young SARS-CoV-2-Positive Chinese Travelers Returning to Shanghai. *Front Public Health* 2020; **8**: 368.

366 Yang N, Shen Y, Shi C, *et al.* In-flight transmission cluster of COVID-19: a retrospective case series. *Infect Dis* 2020; : 1–11.

367 Ma Y-L, Xia S-Y, Wang M, Zhang S-M, Du W-H, Chen Q. Clinical features of children with SARS-CoV-2 infection: an analysis of 115 cases. *Zhongguo Dang Dai Er Ke Za Zhi* 2020; **22**: 290–3.

368 Yassa M, Yirmibes C, Cavusoglu G, *et al.* Outcomes of universal SARS-CoV-2 testing program in pregnant women admitted to hospital and the adjuvant role of lung ultrasound in screening: a prospective cohort study. *J Matern Fetal Neonatal Med* 2020; : 1–7.

369 Yau K, Muller MP, Lin M, *et al.* COVID-19 Outbreak in an Urban Hemodialysis Unit. *Am J Kidney Dis* 2020; published online July 15. DOI:10.1053/j.ajkd.2020.07.001.

370 Cura Yayla BC, Ozsurekci Y, Aykac K, *et al.* Characteristics and Management of Children With COVID-19 in Turkey. *Balkan Med J* 2020; published online Aug 31. DOI:10.4274/balkanmedj.galenos.2020.2020.7.52.

371 Yayla BCC, Aykac K, Ozsurekci Y, Ceyhan M. Characteristics and Management of Children With COVID-19 in a Tertiary Care Hospital in Turkey. *Clin Pediatr* 2020; : 9922820966306.

372 Ye F, Xu S, Rong Z, *et al.* Delivery of infection from asymptomatic carriers of COVID-19 in a familial cluster. *Int J Infect Dis* 2020; **94**: 133–8.

373 Lixia Y, Haibin W, Huaichu L, *et al.* Investigation of a cluster epidemic of COVID-19 in Ningbo. *Chinese Journal of Epidemiology* 2020; **41**: E065–E065.

374 Yılmaz K, Gozupirinççioğlu A, Aktar F, *et al.* Evaluation of the novel coronavirus disease in Turkish children: Preliminary outcomes. *Pediatr Pulmonol* 2020; published online Sept 29. DOI:10.1002/ppul.25095.

375 Yombi JC, De Greef J, Bernard P, Belkhir L. Testing of patients and coronavirus disease 2019 (COVID-19) infection before scheduled deliveries. *J Perinat Med* 2020; **48**: 995–6.

376 Yue H, Bai X, Wang J, *et al.* Clinical characteristics of coronavirus disease 2019 in Gansu province, China. *Ann Palliat Med* 2020; **9**: 1404–12.

377 Zamzuri M ‘ammar IA, Ibrahim FE, Reffien MAM, *et al.* Epidemiological Characteristics of COVID-19 in Seremban, Negeri Sembilan, Malaysia. *Open Access Maced J Med Sci* 2020; **8**: 471–5.

378 Zhan T, Liu M, Tang Y, *et al.* Retrospective analysis of clinical characteristics of 405 patients with COVID-19. *J Int Med Res* 2020; **48**: 300060520949039.

379 Zhang J, Tian S, Lou J, Chen Y. Familial cluster of COVID-19 infection from an asymptomatic. *Crit Care* 2020; **24**: 119.

380 Zhang W, Cheng W, Luo L, *et al.* Secondary Transmission of Coronavirus Disease from Presymptomatic Persons, China. *Emerg Infect Dis* 2020; **26**: 1924–6.

381 Zhang H-J, Su Y-Y, Xu S-L, *et al.* Asymptomatic and symptomatic SARS-CoV-2 infections in close contacts of COVID-19 patients: a seroepidemiological study. *Clin Infect Dis* 2020; published online June 16. DOI:10.1093/cid/ciaa771.

382 Zhang L, Huang S. Clinical Features of 33 Cases in Children Infected With SARS-CoV-2 in Anhui Province, China-A Multi-Center Retrospective Cohort Study. *Front Public Health* 2020; **8**: 255.

383 Zhang H, Chen R, Chen J, Chen B. COVID-19 Transmission Within a Family Cluster in Yancheng, China. *Front Med* 2020; **7**: 387.

384 Zhang S, Guo M, Wu F, *et al.* Factors associated with asymptomatic infection in health-care workers with severe acute respiratory syndrome coronavirus 2 infection in Wuhan, China: a multicentre retrospective cohort study. *Clin Microbiol Infect* 2020; published online Sept 7. DOI:10.1016/j.cmi.2020.08.038.

385 Zhang H, Hong C, Zheng Q, *et al.* A multi-family cluster of COVID-19 associated with asymptomatic and pre-symptomatic transmission in Jixi City, Heilongjiang, China, 2020. *Emerg Microbes Infect* 2020; **9**: 2509–14.

386 Zhao D, Wang M, Wang M, *et al.* Asymptomatic infection by SARS-CoV-2 in healthcare workers: a study in a large teaching hospital in Wuhan, China. *Int J Infect Dis* 2020; published online Aug 3. DOI:10.1016/j.ijid.2020.07.082.

387 Zheng X, Luo S, Sun Y, *et al.* Asymptomatic patients and asymptomatic phases of Coronavirus Disease 2019 (COVID-19): a population-based surveillance study. *Natl Sci Rev* 2020; published online June 23. DOI:10.1093/nsr/nwaa141.

388 Deng ZQ, Xia W, Fan YB, *et al.* Analysis on transmission chain of a cluster epidemic of COVID-19, Nanchang. *Zhonghua Liu Xing Bing Xue Za Zhi* 2020; **41**: 1420–3.

389 周昀, Yun Z, 杨根东, *et al.* 婴幼儿2019冠状病毒病的临床特点及胸部CT表现. 2020; published online March 15. DOI:10.7499/j.issn.1008-8830.2020.03.007.

390 Zou L, Ruan F, Huang M, *et al.* SARS-CoV-2 Viral Load in Upper Respiratory Specimens of Infected Patients. *N Engl J Med* 2020; **382**: 1177–9.s
